# A novel m^6^A reader Prrc2a controls oligodendroglial specification and myelination

**DOI:** 10.1038/s41422-018-0113-8

**Published:** 2018-12-04

**Authors:** Rong Wu, Ang Li, Baofa Sun, Jian-Guang Sun, Jinhua Zhang, Ting Zhang, Yusheng Chen, Yujie Xiao, Yuhao Gao, Qingyang Zhang, Jun Ma, Xin Yang, Yajin Liao, Wei-Yi Lai, Xiaolong Qi, Shukun Wang, Yousheng Shu, Hai-Lin Wang, Fengchao Wang, Yun-Gui Yang, Zengqiang Yuan

**Affiliations:** 1 The Brain Science Center, Beijing Institute of Basic Medical Sciences, 100850 Beijing, China; 2 Key Laboratory of Genomic and Precision Medicine, Collaborative Innovation Center of Genetics and Development, Beijing Institute of Genomics, Chinese Academy of Sciences, 100101 Beijing, China; 3 School of Life Science, University of Chinese Academy of Sciences, 100049 Beijing, China; 4 Institute of Biophysics, Chinese Academy of Sciences, 100101 Beijing, China; 5 The College of Life Science and Bioengineering, Beijing Jiaotong University, Beijing, China; 6 State Key Laboratory of Cognitive Neuroscience and Learning, IDG/McGovern Institute for Brain Research, School of Brain and Cognitive Sciences, the Collaborative Innovation Center for Brain Science, Beijing Normal University, 100875 Beijing, China; 7 State Key Laboratory of Environmental Chemistry and Ecotoxicology, Research Center for Eco-Environmental Sciences, 100085 Beijing, China; 8 National Institute of Biological Sciences, Beijing, China; 9 Institute for Stem Cell and Regeneration, Chinese Academy of Sciences, 100101 Beijing, China; 10 Center of Alzheimer’s Disease, Beijing Institute for Brain Disorders, Beijing, China

**Keywords:** RNA modification, Mechanisms of disease

## Abstract

While *N*^6^-methyladenosine (m^6^A), the most abundant internal modification in eukaryotic mRNA, is linked to cell differentiation and tissue development, the biological significance of m^6^A modification in mammalian glial development remains unknown. Here, we identify a novel m^6^A reader, Prrc2a (Proline rich coiled-coil 2 A), which controls oligodendrocyte specification and myelination. *Nestin*-Cre-mediated knockout of Prrc2a induces significant hypomyelination, decreased lifespan, as well as locomotive and cognitive defects in a mouse model. Further analyses reveal that Prrc2a is involved in oligodendrocyte progenitor cells (OPCs) proliferation and oligodendrocyte fate determination. Accordingly, oligodendroglial-lineage specific deletion of *Prrc2a* causes a similar phenotype of *Nestin*-Cre-mediated deletion. Combining transcriptome-wide RNA-seq, m^6^A-RIP-seq and Prrc2a RIP-seq analysis, we find that *Olig2* is a critical downstream target gene of Prrc2a in oligodendrocyte development. Furthermore, Prrc2a stabilizes *Olig2* mRNA through binding to a consensus GGACU motif in the *Olig2* CDS (coding sequence) in an m^6^A-dependent manner. Interestingly, we also find that the m^6^A demethylase, Fto, erases the m^6^A modification of *Olig2* mRNA and promotes its degradation. Together, our results indicate that Prrc2a plays an important role in oligodendrocyte specification through functioning as a novel m^6^A reader. These findings suggest a new avenue for the development of therapeutic strategies for hypomyelination-related neurological diseases.

## Introduction

*N*^6^-methyladenosine (m^6^A) is the most abundant internal modification of mRNA in eukaryotes.^1–3^ m^6^A modification is dynamically regulated by a series of enzymes including m^6^A methyltransferases, demethylases, and m^6^A-specific binding proteins, which respectively write, erase, and recognize the m^6^A mark.^4–8^ The function of the methyltransferase METTL3 has been previously studied in the context of stem cell pluripotency and cell differentiation,^9,10^ whereas the demethylases FTO and ALKBH5 serve as regulatory factors in energy homeostasis, adipocyte differentiation, tumorigenesis, and fertility in mice.^11–15^ Recently, several m^6^A-specific readers including YTH-domain containing family proteins, hnRNP proteins (hnRNPA2B1, hnRNPC) and IGF2BPs have been identified.^2,16–24^ These m^6^A readers modulate RNA stability, translation, and splicing as well as RNA-protein interactions.^16,18–23,25^

RNA binding proteins (RBPs) are highly expressed in the brain and serve to regulate alternative splicing, transport, localization, stability, and translation of RNAs during development.^26^ The m^6^A RNA modification is abundant in the brain,^3^ and recent studies have demonstrated the role of the m^6^A modification in *Drosophila* neural function and mammalian neurogenesis.^27–32^ The m^6^A mRNA modification has also been proven critical for glioblastoma stem cell (GSC) self-renewal and tumorigenesis^14,15^ suggesting the functional importance of the m^6^A mRNA methylation in glial cells. Despite that multiple m^6^A readers are identified, none of them has been reported to impact glial development.

Glial cells make up at least 50% of the cells in the brain and oligodendrocytes, a subclass of glial cells, are necessary for CNS myelination.^33,34^ Although oligodendrocytes are indispensable for normal brain development and function, the molecular mechanisms of oligodendroglial specification are incompletely understood.

Here, we identify a novel m^6^A-specific binding protein, Prrc2a, in neural cells, and importantly, we find that Prrc2a deficiency in the brain leads to hypomyelination by affecting oligodendroglial specification. Combining transcriptome-wide RNA-seq, m^6^A-seq and Prrc2a RIP-seq analyses, we find that Prrc2a directly regulates *Olig2* expression in an m^6^A-dependent manner in vitro and in vivo. Collectively, our study elucidates a new post-transcriptional regulation mechanism in oligodendroglial specification and myelination.

## Results

### Prrc2a is a novel m^6^A reader

**Fig. 1:**
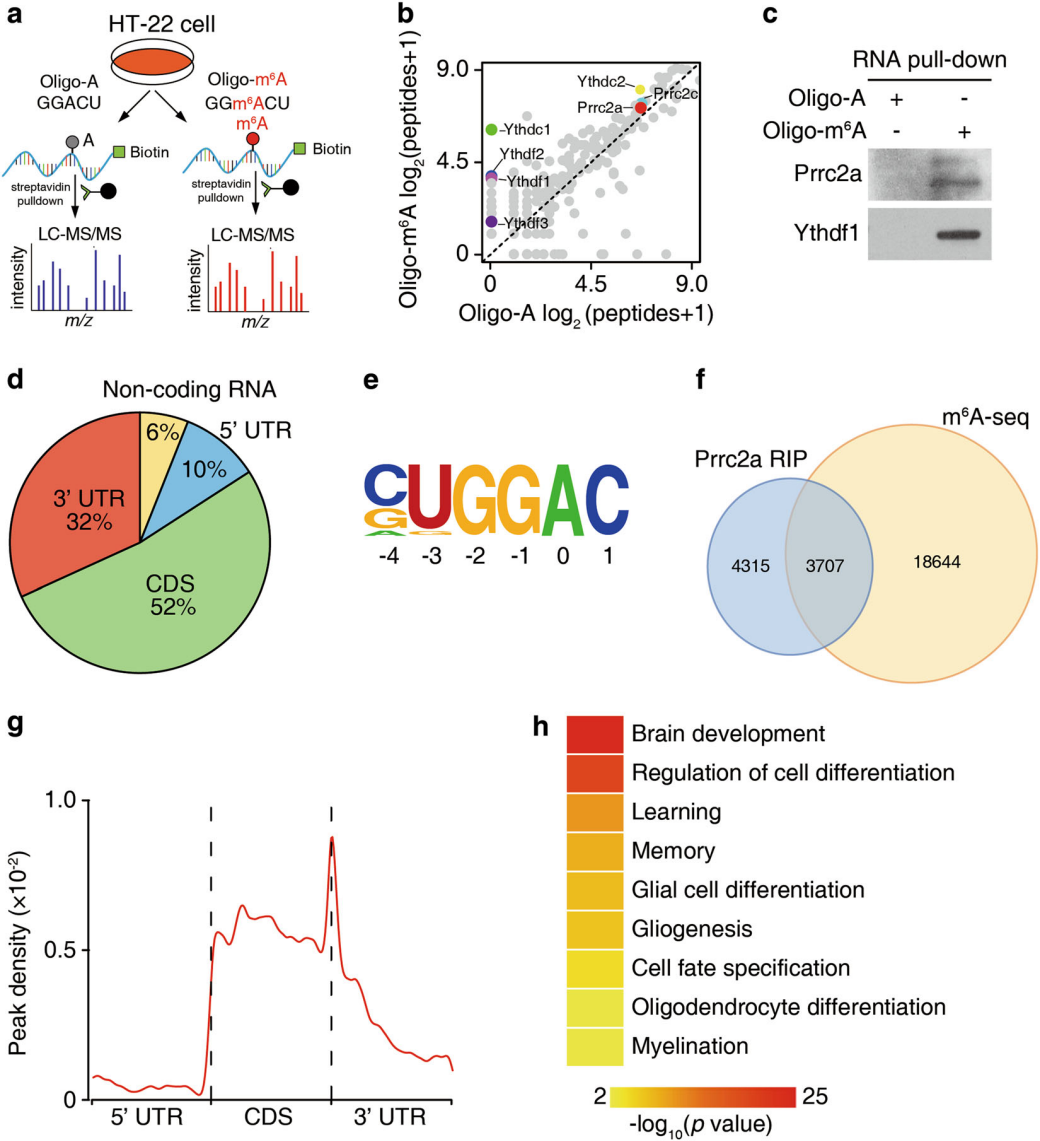
Prrc2a is a novel m^6^A reader. **a** Schematic illustration of m^6^A binding protein screening. **b** Scatter plot of proteins bound to Oligo-m^6^A vs Oligo-A RNA oligos. The plot was based on the average peptide numbers of proteins detected in two replicates. Enriched Prrc2a, Prrc2c, and YTH-domain containing proteins were highlighted (see also Supplementary information, Table S1). **c** Western blotting showing Ythdf1 and Prrc2a pulled down with an m^6^A-containing RNA probe. **d** Pie chart depicting the distribution of Prrc2a-binding peaks. **e** Binding motif identified by HOMER with Prrc2a-binding peaks (*p* = 1e-46). **f** Overlap of Prrc2a-binding peaks and m^6^A-containing peaks. **g** Distribution of m^6^A-containing Prrc2a peaks across the length of mRNA. 5′ UTR, CDS, and 3′ UTR were each binned into regions spanning 1% of their total length, and the percentages of m^6^A-containing Prrc2a peaks that fall within each bin were determined. The moving averages of m^6^A-containing Prrc2a peak percentage are shown. **h** Representative Gene Ontology (GO) terms of the biological process categories enriched in transcripts with both Prrc2a-binding and m^6^A peaks. Gene ontology (GO) analysis was performed using the DAVID bioinformatics database. GO classification for cellular component, biological process, and molecular function were performed with default settings

To decipher the role of the m^6^A modification in neural development and neurological disorders, we first sought to define whether there were new m^6^A-specific binding proteins in neural cells. By using methylated RNA bait containing the known consensus sites of G(m^6^A)C vs unmethylated control in cell lysates of HT-22 cells (a neuronal cell line), we identified that Prrc2a (Proline rich coiled-coil 2 A) and Prrc2c (Proline rich coiled-coil 2 C) were potential m^6^A binding proteins (Fig. 1a–c, Supplementary information, Fig. S1a and b, Supplementary information, table S1). Interestingly, *Prrc2a* was more expressed in all types of neural cells than *Prrc2c* based on the brain-seq database^35^ (Supplementary information, Fig. S1c). Furthermore, we found that Prrc2a was highly expressed in oligodendrocyte precursor cells (OPCs) in cultured neural cells (Supplementary information, Fig. S1d).

*Prrc2a* encodes a large proline-rich protein and is within human major histocompatibility complex III region.^36^ However, little is known about the pathophysiological functions of Prrc2a in the nervous system. Full length Prrc2a is mostly located in cytoplasm (Supplementary information, Fig. S1e). The P2 fragment of Prrc2a that contains the enriched glycine, arginine and glutamic acid (here named GRE domain) was found to specifically bind RNA in a photoactivatable ribonucleotide crosslinking and immunoprecipitation (PAR-CLIP) assay (Supplementary information, Fig. S1e and f). Further gel-shift assays revealed that recombinant Prrc2a-p2 protein had a higher binding affinity to methylated probes compared to unmethylated controls (Supplementary information, Fig. S1g and h). Additionally, we found that the recombinant Prrc2a-p2 preferred to bind m^6^A-containing RNAs by using LC-MS/MS (Supplementary information, Fig. S1i).

We next performed Prrc2a RIP-seq and m^6^A-seq in brain samples to further demonstrate that Prrc2a selectively binds m^6^A-containing RNA (Supplementary information, Fig. S1j and k). A total of 8022 Prrc2a binding peaks within 2858 genes were identified in both biological replicates, and most of them (2,646/2,858) were located in mRNAs, similar to the typical m^6^A distribution (Fig. 1d). Prrc2a predominantly targeted the coding regions and 3′ UTR of mRNA transcripts (Fig. 1d), which is consistent with the previously reported pattern of m^6^A peaks. Furthermore, a GGAC motif, coinciding with the consensus m^6^A motif, was enriched in Prrc2a binding peaks (Fig. 1e).

The distribution features of m^6^A peaks identified in whole brain were consistent with previous reports, suggesting the reliability of our m^6^A-seq data (Supplementary information, Fig. S1l-n). We overlaid the Prrc2a binding peaks with m^6^A peaks and found that 46% (3707 out of 8022) of the peaks of Prrc2a overlapped with m^6^A peaks, suggesting an enrichment of m^6^A at Prrc2a binding regions (Fig. 1f and Supplementary information, Fig. S1o). Among 3707 Prrc2a peaks that overlapped with m^6^A, enrichment occurred around stop codons, resembling the distribution of the m^6^A modification (Fig. 1g). To gain insight into the potential function of Prrc2a, we performed gene ontology (GO) enrichment analysis on Prrc2a target mRNAs with m^6^A peaks and found that they were involved in various functions, including nervous system development, cell differentiation, and myelination (Fig. 1h), indicating Prrc2a may be involved in glial development. Taken together, we conclude that Prrc2a is a novel m^6^A reader and may regulate brain functioning.

### Prrc2a deficiency in brain leads to hypomyelination

**Fig. 2:**
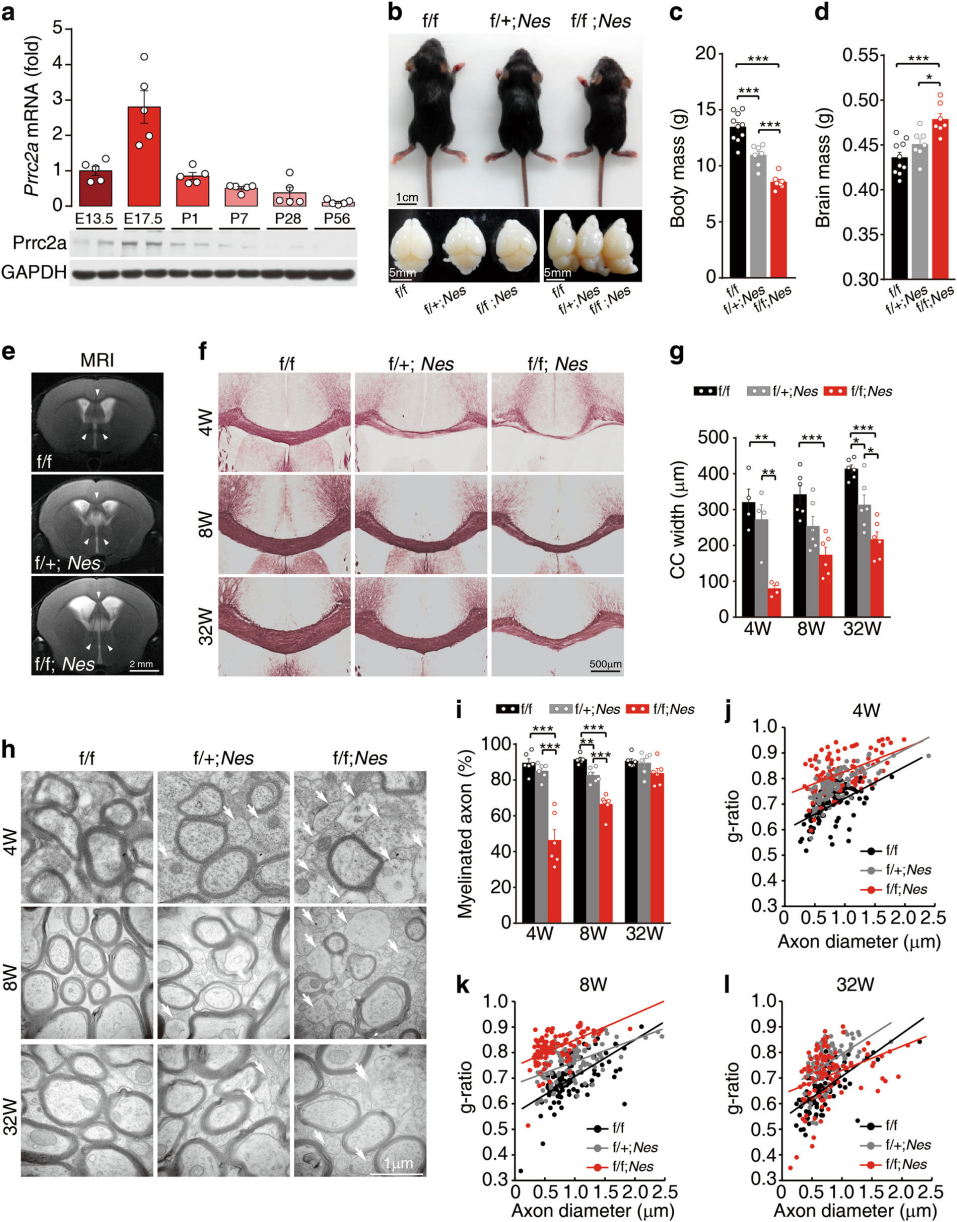
*Prrc2a*^*f/f*^*;Nestin*^*Cre+/-*^ mice display hypomyelination at early stage. **a** The expression pattern of Prrc2a in the whole brain during brain development. The upper panel shows mRNA level of Prrc2a and the bottom panel shows the protein level of Prrc2a. **b** Representative pictures of *Prrc2a*^*f/f*^*;Nestin*^*Cre+/-*^ mice and brain at P14. **c** Body mass of male mice with indicated genotypes at P28 (one-way ANOVA followed Tukey test, ***P < 0.001, f/f *n* = 10, f/ + ;*Nes* *n* = 7, f/f;*Nes* *n* = 7). **d** The whole wet brain mass of male mice with indicated genotypes at P28 (one-way ANOVA followed Tukey test, *P < 0.05, ***P < 0.001, f/f *n* = 10, f/ + ;*Nes* *n* = 7, f/f;*Nes* *n* = 7). **e** T2-weighted magnetic response imaging of mice with indicated genotypes at P56. **f** Black-gold staining of brain slices from 4-, 8-, and 32-week-old mice of indicated genotypes. **g**The quantification of corpus callosum width at the midline of 4-, 8-, and 32-week-old mice (one-way ANOVA followed Tukey test, *P < 0.05, **P < 0.01, ***P < 0.001, 4w: *n* = 4 each group; 8w: *n* = 6 each group; 32w: *n* = 6 each group). **h** Representative transmission electron micrographs (TEM) of the myelin fibers in the corpus callosum showed reduced myelination, naked axons, and vacuolation from 4-, 8-, and 32-week-old mice with indicated genotypes, the white arrowheads indicated the naked axons. **i** The percentage of myelinated axons in the corpus callosum from 4-, 8-, and 32-week-old mice with indicated genotypes (one-way ANOVA followed Tukey test, **P < 0.01, ***P < 0.001, *n* = 6 per group). **j–l** Scatterplots of the myelin g ratios (diameter of axon/diameter of entire fiber) in the corpus callosum of 4-, 8-, and 32-week-old mice with indicated genotypes (general linear model and ANCOVA analysis, 4w (**j**): f/f *vs*. f/ + *;Nes*, *P* < 0.001; f/f *vs*. f/f*;Nes*, *P* < 0.001; f/ + *;Nes vs*. f/f*;Nes*, *P* < 0.001; 8w(**k**): f/f *vs*. f/ + *;Nes*, *P* < 0.001; f/f *vs*. f/f*;Nes*, *P* < 0.001; f/ + *;Nes vs*. f/f*;Nes*, *P* < 0.001; 32w(**l**): f/f *vs*. f/ + *;Nes*, *P* < 0.001; f/f *vs*. f/f*;Nes*, *P* < 0.001; f/ + *;Nes vs*. f/f*;Nes*, *P* < 0.001; More than 100 axons from each genotype and time points were analyzed)

To explore the role of Prrc2a in the nervous system, we first examined the expression pattern of Prrc2a in the brain. We found that Prrc2a was broadly expressed with high expression levels in the embryonic stage (Fig. 2a and Supplementary information, Fig. S2a). Specifically, Prrc2a was mainly expressed in Pdgfrα- or NG2- positive cells in white matter (corpus callosum) and the expression level of Prrc2a decreased at 8-week-old (Supplementary information, Fig. 2b), suggesting Prrc2a might play an important role during neural development. To address this, *Nestin-Cre* transgenic mice were crossed with *Prrc2a*^*f/f*^ mice. We found that *Nestin*-Cre significantly ablated Prrc2a expression in brain and different neural cell types (Supplementary information, Fig. S2c-e) and specific loss of *Prrc2a* in the brain led to development delay, lower body mass, increased brain mass, enlarged lateral ventricles, and abnormal brain structures (Fig. 2b–d and Supplementary information, Fig.S2f). However, there was no significant alteration of neuron number in Prrc2a conditional knockout mice (Supplementary information, Fig. S2g-i). Further T2-weighted magnetic resonance imaging (MRI) analysis showed increased brain volume, enlarged lateral ventricles, and increased signal intensity of the corpus callosum in Prrc2a-deficient mice (Fig. 2e). Since white matter fibers of the corpus callosum are mainly composed of axons and the enveloping myelin, we hypothesized that Prrc2a deficiency may lead to widespread myelination defects in the brain. As expected, *Prrc2a*^*f/f*^*;Nestin*^*Cre+/-*^ mice showed severe hypomyelination in the corpus callosum at young (4-week-old) and adult stages (8-and 32-week-old) as indicated by black-gold staining (Fig. 2f, g). Transmission Electron Microscopy (TEM) analysis further demonstrated a markedly reduced percentage of myelinated axons and myelin thickness in different brain regions from *Prrc2a*^*f/f*^*;Nestin*^*Cre+/-*^ mice (Fig. 2h–l and Supplementary information, Fig. S2j and k). Functionally, we also observed that Prrc2a deficiency significantly impaired callosal conduction velocity (Supplementary information, Fig. S2l-n). Collectively, ablation of Prrc2a in brain induces hypomyelination.

**Fig. 3:**
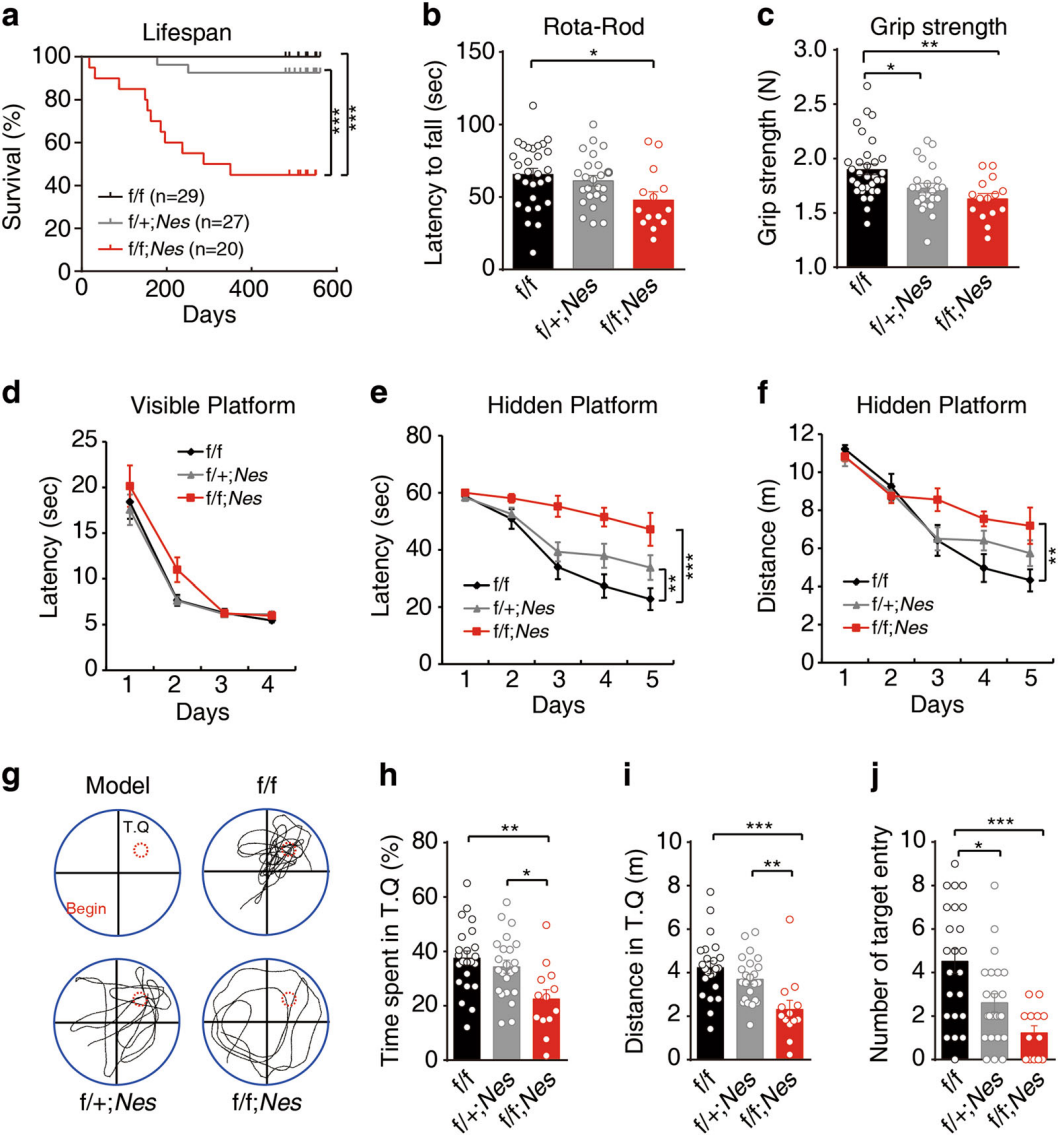
Effects of Prrc2a on longevity, motor behavior, and cognition. **a** Kaplan–Meier survival curves. Statistical significance was determined by the log rank test (***P < 0.001; f/f *n* = 29, f/ + ;*Nes* *n* = 27, f/f;*Nes* *n* = 20). **b** The latency of 3-month-old mice on the Rota-Rod (one-way ANOVA followed Tukey test, *P < 0.05, f/f, *n* = 29, f/ + ; *Nes* *n* = 24, f/f; *Nes* *n* = 14). **c** Grip strength analysis of 3-month-old mice with indicated genotypes (one-way ANOVA followed Tukey test, *P < 0.05, **P < 0.01, f/f, *n* = 32, f/ + ; *Nes* *n* = 24, f/f; *Nes* *n* = 15). **d** The mean escape latency (± SEM) for mice to reach the platform in the visible version of the water maze is plotted against the day of the experiment (f/f vs. f/ + ; *Nes*, *P* = 0.8972, *F* = 0.01688; f/f vs. f/f; *Nes*, *P* = 0.0999, *F* = 2.860; f/ + ; *Nes* vs. f/f; *Nes*, *P* = 0.0674, *F* = 3.570. Two-way ANOVA followed by Bonferroni test. f/f *n* = 23, f/ + ;*Nes* *n* = 23, f/f;*Nes* *n* = 13). **e** The mean escape latency ( ± SEM) for mice to reach the platform in the hidden version of the water maze is plotted against the day of the experiment (f/f vs. f/ + ; *Nes*, *P* = 0.0630, *F* = 3.639; f/f vs. f/f; *Nes*, *P* < 0.0001, *F* = 23.10; f/ + ; *Nes* vs. f/f; *Nes*, *P* = 0.0050, *F* = 8.992. Two-way ANOVA followed by Bonferroni test. f/f *n* = 23, f/ + ;*Nes* *n* = 23, f/f;*Nes* *n* = 13). **f** The mean traveled distance ( ± SEM) for mice to reach the platform in the hidden version of the water maze is plotted against the day of the experiment (f/f vs. f/ + ; *Nes*, *P* = 0.3979, *F* = 0.7289; f/f vs. f/f; *Nes*, *P* = 0.0097, *F* = 7.509; f/ + ; *Nes* vs. f/f; *Nes*, *P* = 0.0615, *F* = 3.740. Two-way ANOVA followed by Bonferroni test. f/f *n* = 23, f/ + ;*Nes* *n* = 23, f/f;*Nes* *n* = 13). **g** Probe trial was performed 24 h after the last training session by removing the platform. Probe model and representative vertical views of the tracks of mice with indicated genotypes were shown. **h** Time spent in the target quadrant during probe trial (one-way ANOVA followed Tukey test, *P < 0.05, **P < 0.01, f/f, *n* = 23, f/ + ; *Nes* *n* = 23, f/f; *Nes* *n* = 13). **i** Traveled distance in the target quadrant during probe trial (one-way ANOVA followed Tukey test, **P < 0.01, ***P < 0.001, f/f, *n* = 23, f/ + ; *Nes* *n* = 23, f/f; *Nes* *n* = 13). **j** The number of platform crossing from the same group of mice tested in the probe trial (one-way ANOVA followed Tukey test, *P < 0.05, ***P < 0.001, f/f, *n* = 23, f/ + ; *Nes* *n* = 23, f/f; *Nes* *n* = 13). The water maze behavior was tested in 6-month-old mice

Myelin dysfunction has a profound effect on locomotion, cognition, and lifespan.^37^ Besides a higher mortality in *Prrc2a*^*f/f*^*;Nestin*^*Cre+/-*^ mice (Fig. 3a), overt locomotive defects and a 15% reduction in grip strength were also observed in *Prrc2a*^*f/f*^*;Nestin*^*Cre+/-*^ mice (Fig. 3b, c). We also found learning and memory defects in *Prrc2a*^*f/f*^*;Nestin*^*Cre+/-*^ mice (Fig. 3d–j). Taken together, *Prrc2a* deficiency induces hypomyelination, impaired locomotive, and cognitive disability.

### Prrc2a modulates oligodendrocyte lineage specification

**Fig. 4:**
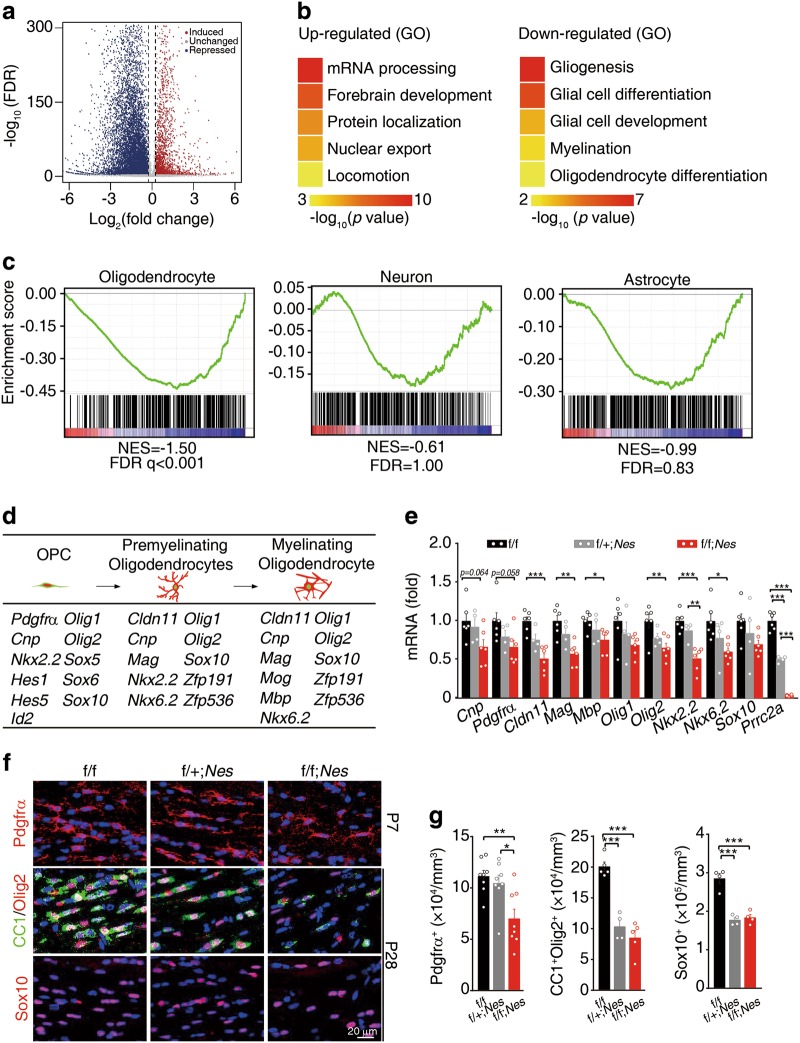
Prrc2a deficiency reduces oligodendroglia proliferation. **a** Volcano plot of RNA-seq data shows Prrc2a-regulated genes from brain tissue samples of 4-week-old *Prrc2a*^*f/f*^*; Nestin*^*Cre+/-*^ vs. control mice. **b** Representative Gene Ontology (GO) terms of the biological process categories enriched in transcripts with upregulated (left) or downregulated (right) expressions from *Prrc2a*^*f/f*^*; Nestin*^*Cre+/-*^ vs. control samples. **c** GSEA plots evaluating the changes in oligodendroglial lineage-specific genes, the neuronal genes and astrocytic genes in *Prrc2a*^*f/f*^*; Nestin*^*Cre+/-*^ vs. control brain tissue samples. Note that FDR < 0.25 is statistically significant for GSEA analysis: www.broadinstitute.org/gsea/doc/GSEAUserGuideFrame.html. (see also Supplementary information, Table S4-6). **d** Schematic cartoon of oligodendroglia developmental stages: OPC, premyelinating, and ultimately myelinating oligodendroglia. The below table shows the down-regulated DEGs that control oligodendroglia-specific stage development. **e** Relative gene expression in hippocampus tissue from 4-week-old mice with indicated genotypes (one-way ANOVA followed Tukey test, *P < 0.05, **P < 0.01, ***P < 0.001, f/f, *n* = 6; f/ + ; *Nes*, *n* = 4; f/f; *Nes*, *n* = 6). **f** Immunostainings of Pdgfrα (P7), CC1/Olig2, or Sox10 (P28) in corpus callosum from mice with indicated genotypes. The quantification of Pdgfrα^+^, CC1^+^ Olig2^+^, or Sox10^+^ cells was shown in (**g**) (one-way ANOVA followed Tukey test, *P < 0.05, **P < 0.01, ***P < 0.001, Pdgfrα^+^ cells, n = 8 each group, CC1^+^ Olig2^+^ cells, f/f, *n* = 5, f/ + ; *Nes* *n* = 4, f/f; *Nes* *n* = 5, Sox10^+^ cells, *n* = 4 each group)

To systemically investigate the mechanism underlying the development of hypomyelination in the context of Prrc2a deficiency, we performed RNA sequencing (RNA-seq) analysis on control and *Prrc2a*^*f/f*^*;Nestin*^*Cre+/-*^ mouse brains and found that Prrc2a deficiency had profound effects on the gene expression landscape as thousands of differentially expressed genes (DEGs) were detected (Fig. 4a). Interestingly, we found that the downregulated DEGs were significantly enriched in glial cell differentiation and myelination pathways (Fig. 4b). Among 373 oligodendroglial DEGs, 71 genes were Prrc2a targets (Supplementary information, Fig. S3a and Supplementary information, Table S2-4). Gene set enrichment analyses (GSEA) analysis revealed a marked downregulation of oligodendroglial lineage-specific genes, but not astrocyte- and neuron-related genes in Prrc2a-deficient mice (Fig. 4c and Supplementary information, Table S4-6). Upon further analysis of the gene expression, we found that a panel of oligodendroglial marker genes and oligodendroglial lineage specification-related transcription factors, such as *Cnp*, *Cldn11, Mbp, Olig1*, *Olig2*, *Nkx2.2*, and *Sox10*, were downregulated upon Prrc2a deletion (Fig. 4d and Supplementary information, Fig. S3b). The downregulation of oligodendroglial lineage-specific genes was confirmed by RT-qPCR (Fig. 4e), which validated the reliability of our transcriptome-wide RNA-seq data. Consistent with the transcriptional changes, the protein levels of Olig2 (an oligodendroglial specification-related transcription factor) and Mbp (myelin sheath structural protein) were also reduced in Prrc2a-deficient mice (Supplementary information, Fig. S3c). The dramatic reduction of oligodendroglial lineage-related transcripts and proteins suggested a reduced oligodendroglial population. We found that Prrc2a deficiency significantly reduced the numbers of Pdgfrα^+^ (OPCs), CC1^+^Oilg2^+^ (mature oligodendrocytes), or Sox10^+^ cells (Fig. 4f, g, Supplementary information, Fig. S3d and e). Taken together, Prrc2a deletion reduces the expression of oligendendroglial genes and oligodendroglia population.

**Fig. 5:**
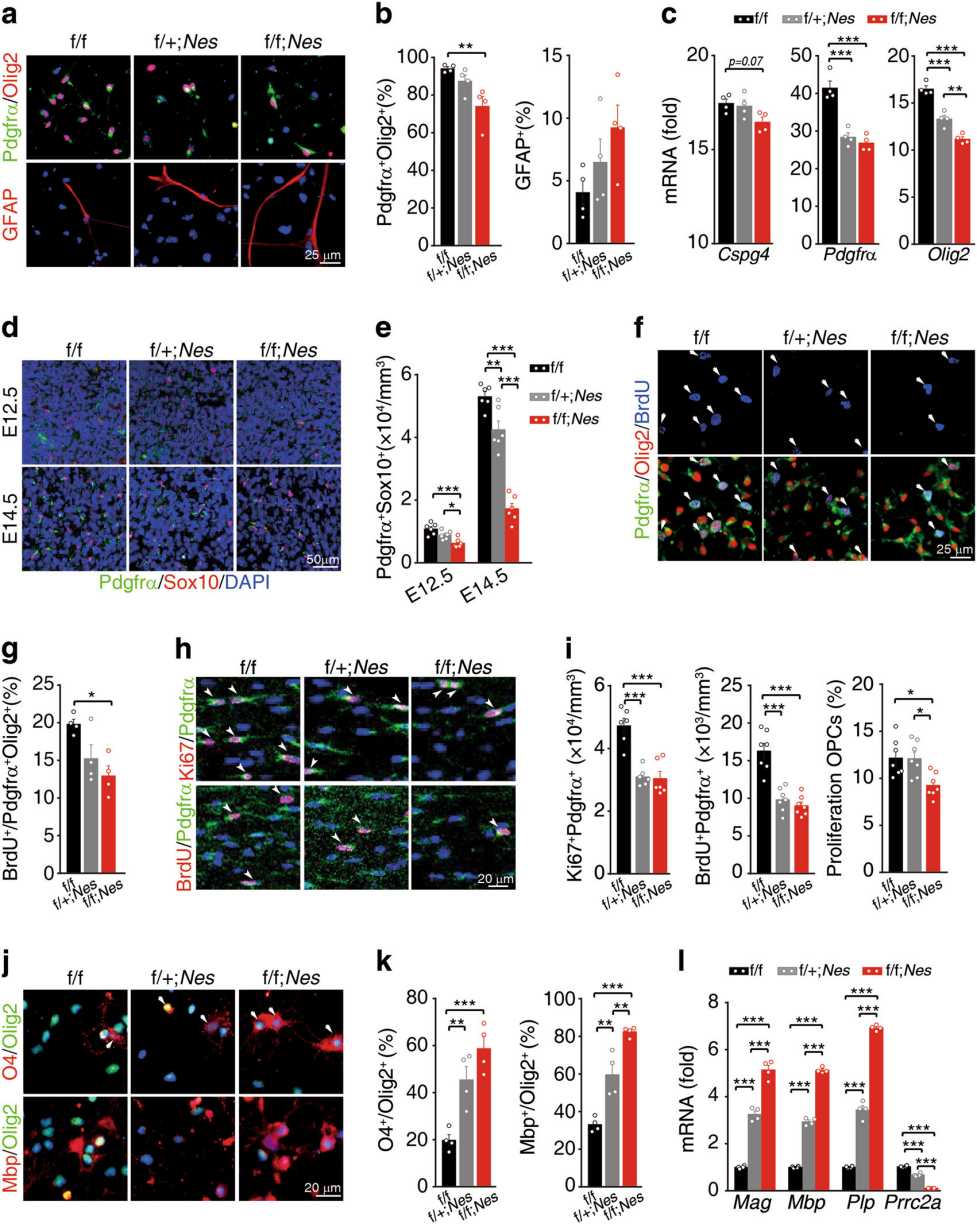
Prrc2a modulates oligodendroglia fate determination, proliferation, and differentiation in vitro. **a** Immunostainings of Pdgfrα/Olig2 or GFAP in oligosphere-derived cells 14 days postBl04-CM treatment from mice with indicated genotypes. **b** Quantification of the percentage of Pdgfrα^+^Olig2^+^ and GFAP^+^ cells from mice with indicated genotypes (one-way ANOVA followed Tukey test, **P < 0.01, *n* = 4 each group). **c** Gene expressions during neurosphere to oligosphere transformation in cells 14 days post B104-CM treatment. The gene expressions were normalized to those in wild-type neural stem cells (one-way ANOVA followed Tukey test, **P < 0.01, ***P < 0.001, *n* = 4 per group). **d** Immunostainings of Pdgfrα and Sox10 in spinal cord from mice with indicated genotypes at E12.5 and E14.5. **e** The quantification of the number of OPCs (Pdgfrα and Sox10 double positive) in spinal cord from mice with indicated genotypes at E12.5 and E14.5 (one-way ANOVA followed Tukey test, *P < 0.05, **P < 0.01, ***P < 0.001, *n* = 6 per group). **f** Immunostainings of Pdgfrα/Olig2/BrdU in the cultured OPCs after incubation with BrdU (50 μg/ml) for 2 h. The bar graph (**g**) depicts the quantification of the percentage of BrdU^+^ Pdgfrα^+^ Olig2^+^ cells (one-way ANOVA followed Tukey test, *P < 0.05, *n* = 4 per group). **h** BrdU (50 mg/kg) was intraperitoneally injected into P6 mice. 2 h later, the mice were sacrificed and the brain sections of corpus callosum were immunostained with anti-Pdgfrα and anti-Ki67 (upper panel) or anti-BrdU (bottom panel) antibodies. Arrowheads indicate the proliferating OPCs (Pdgfrα and Ki67/BrdU double-positive cells). **i**The quantification of the number and percentage of proliferating OPCs from mice with indicated genotypes (one-way ANOVA followed Tukey test, *P < 0.05, ***P < 0.001, Pdgfrα^+^Ki67^+^: *n* = 6 each group; Pdgfrα^+^BrdU^+^: *n* = 7 each group; Proliferation OPCs Pdgfrα^+^BrdU^+^/Pdgfrα^+^: *n* = 7 each group). **j** Immunostainings of O4/Olig2 and Mbp/Olig2 in oligodendrocytes after 3 days of T3 treatment. The bar graph (**k**) depicts the quantification of the percentage of O4^+^ Olig2^+^ and Mbp^+^Olig2^+^ cells (one-way ANOVA followed Tukey test, **P < 0.01, ***P < 0.001, *n* = 4 per group). **l** Gene expressions of T3-induced differentiated oligodendrocytes from mice with indicated genotypes (one-way ANOVA followed Tukey test, ***P < 0.001, *n* = 4 per group)

To clarify how Prrc2a deficiency reduced oligodendroglial population, we first examined whether Prrc2a deficiency reduced OPC numbers through controlling OPC fate determination. NSCs (neural stem cells) were successfully differentiated to OPCs by Bl04 cell conditional medium (B104-CM) treatment in vitro^38^ (Supplementary information, Fig. S4a-c). Interestingly, the expression levels of Prrc2a were increased during this differentiation process (Supplementary information, Fig. S4b). Furthermore, we found fewer OPCs (Pdgfrα^+^/Olig2^+^) and a trend for more astrocytes (GFAP^+^) in Prrc2a-deficient cells (Fig. 5a–c and Supplementary information, Fig. S4d). To explore whether Prrc2a modulates OPCs fate determination in vivo, we analyzed the onset of oligodendroglial specification in the spinal cord. We found that Prrc2a deficiency significantly reduced the number of Pdgfrα^+^/Sox10^+^ double-positive cells in the spinal cord at E12.5 (Fig. 5d, e). Notably, the reduction of OPCs was more obvert in Prrc2a deficiency group at E14.5 (Fig. 5d, e), suggesting that Prrc2a deficiency might also reduce the proliferation of OPCs. In vitro BrdU labeling showed that the proliferation of OPCs was significantly decreased in Prrc2a-deficient cells (Fig. 5f, g). Furthermore, in vivo assays show that Prrc2a deletion led to a significant reduction of OPCs proliferation in corpus callosum (Fig. 5h, i). Surprisingly, we noticed that Prrc2a deficiency actually promoted OPC differentiation in vitro (Fig. 5j–l). Taken together, Prrc2a deficiency inhibits OPCs generation and proliferation, while promoting oligodendrocyte differentiation.

Prrc2a deletion increased astrocyte generation (Fig. 5b and Supplementary information, Fig. S4d) and upregulated parts of astrocytic gene expressions in vivo (Supplementary information, Fig. S3b and c). However, only a slight increase in astrocyte number was observed in Prrc2a deficient mice (Supplementary information, Fig. S4e-h). Further analysis revealed that Prrc2a deficiency significantly reduced the proliferation of astrocytes (Supplementary information, Fig. S4i and j), which could explain why we failed to observe an obvious alteration of astrocyte numbers. Taken together, Prrc2a controls glial cell fate determination and proliferation.

### Prrc2a deletion in oligodendroglial cells induces hypomyelination

**Fig. 6:**
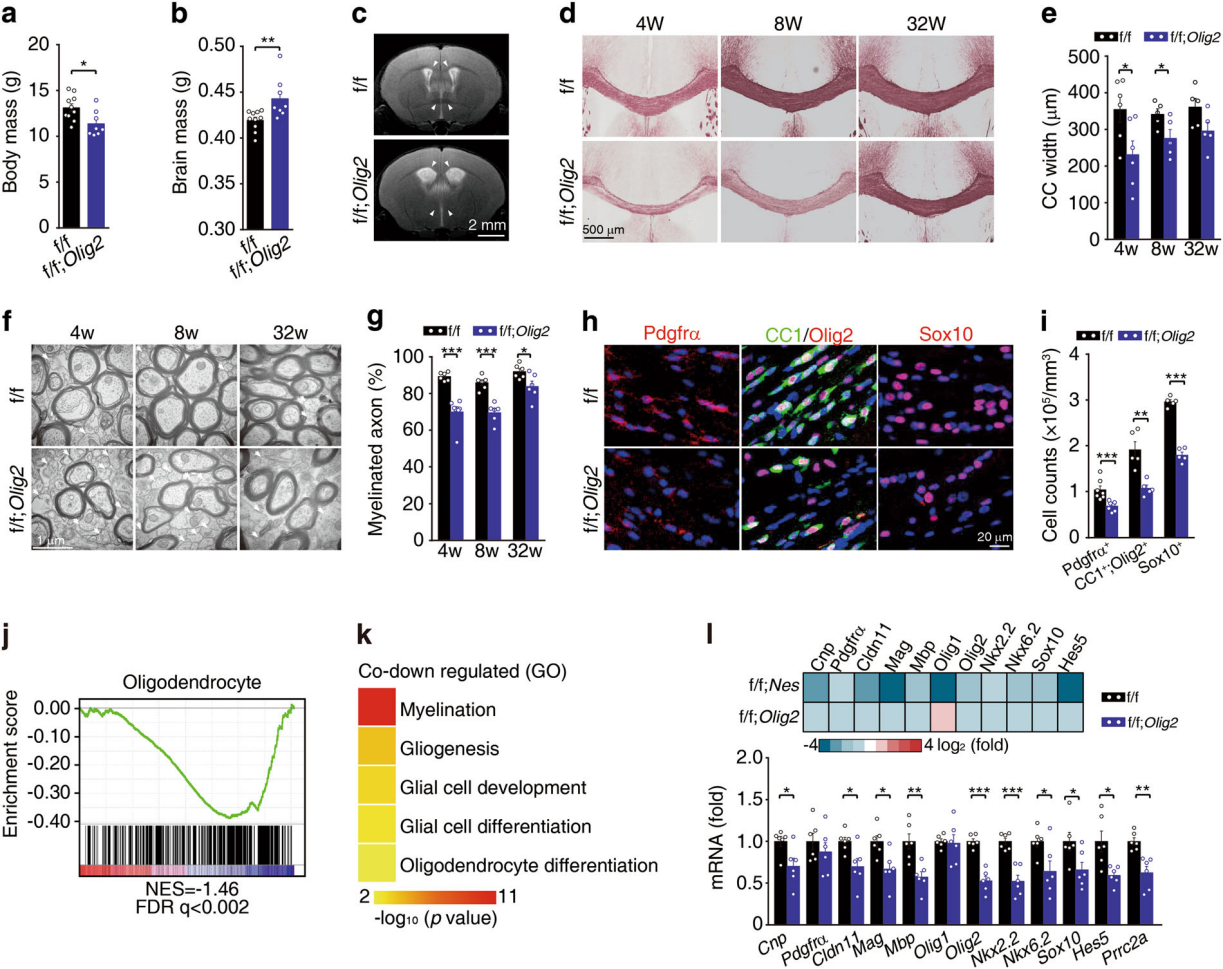
Prrc2a deletion in oligodendroglial lineage induces hypomyelination. **a**, **b** Body and whole wet brain mass of *Prrc2a*^*f/f*^*;Olig2*^*Cre+/-*^ and control mice at P28 (two-tailed unpaired Student’s *t-test*, *P < 0.05, **P < 0.01, f/f, *n* = 10, f/f; *Olig2* *n* = 8). **c** T2-weighted MRI examination of 4-week-old mice with indicated genotypes. **d** Black-gold staining of brain slices from 4-, 8-, and 32-week-old *Prrc2a*^*f/f*^*; Olig2*^*Cre+/-*^ and control mice. **e** The quantification of corpus callosum width at the midline of 4-, 8-, and 32-week-old *Prrc2a*^*f/f*^*; Olig2*^*Cre+/-*^ and control mice (two-tailed unpaired Student’s *t-test*, *P < 0.05, 4w: *n* = 6 each group, 8w: *n* = 5 each group, 32w: *n* = 5 each group). **f** TEM analysis of the myelin fibers in the corpus callosum from 4-, 8-, and 32-week-old *Prrc2a*^*f/f*^*; Olig2*^*Cre+/-*^ and control mice. The white arrowheads indicated the naked axons. **g** The percentage of myelinated axons in the corpus callosum from 4-, 8-, and 32-week-old mice with indicated genotypes (two-tailed unpaired Student’s *t-test*, *P < 0.05, ***P < 0.001, 4w: f/f, *n* = 6, f/f; *Olig2*-Cre *n* = 7, 8w: *n* = 6 each group, 32w: *n* = 6 each group). **h** Immunostainings of Pdgfrα (P7), CC1/Olig2, or Sox10 (P28) in corpus callosum from mice with indicated genotypes. The quantification of Pdgfrα^+^, CC1^+^ Olig2^+^, or Sox10^+^ cells was shown in (**i**) (two-tailed unpaired Student’s *t-test*, **P < 0.01, ***P < 0.001, Pdgfrα ^+^ cells: *n* = 7 per group; CC1^+^ Olig2^+^ cells: *n* = 5 per group, Sox10^+^ cells: *n* = 5 per group). **j** GSEA plots evaluating the changes in oligodendroglia linage specific genes in brain tissue samples from 4-week-old *Prrc2a*^*f/f*^*; Olig2*^*Cre+/-*^ vs. control mice. Note that FDR < 0.25 is statistically significant for GSEA analysis. **k** Representative GO terms of the biological process categories enriched in transcripts with downregulated expressions from *Prrc2a*^*f/f*^*; Nestin*^*Cre+/-*^ and Prrc2af^*/f*^*; Olig2*^*Cre+/-*^ mice. **l** Upper panel shows the heamap of representative DEGs from *Prrc2a*^*f/f*^*; Nestin*^*Cre+/-*^ and Prrc2af^*/f*^*; Olig2*^*Cre+/*^, respectively. Bottom panel shows relative gene expression in hippocampus from indicated groups by RT-qPCR analysis (two-tailed unpaired Student’s *t-test*, *P < 0.05, **P < 0.01, ***P < 0.001, *n* = 6 per group)

To confirm that Prrc2a deficiency-induced hypomyelination is due to the abnormal oligodendroglial lineage development, we constructed the conditional deletion of Prrc2a in oligodendroglia by crossing *Prrc2a*^*f/f*^ mice with *Olig2*-*Cre* mice. In these mice, the expression of Cre recombinase is controlled by endogenous *Olig2* promoter, which led to a haploinsufficience of *Olig2* (Supplementary information, Fig. S5a). Consistence with a previous study,^39^ we found no obvious abnormality of myelin development in brains from *Olig2*-*Cre* mice (Supplementary information, Fig. S5a-c). For *Prrc2a*^*f/f*^*; Olig2*^*Cre+/-*^ mice, *Olig2*-Cre significantly reduced the expression of Prrc2a in Olig2-positive cells (Supplementary information, Fig. S5d). Similar to *Prrc2a*^*f/f*^*; Nestin*^*Cre+/-*^ mice, *Prrc2a*^*f/f*^*; Olig2*^*Cre+/-*^ mice showed remarkable development delay, reduced body mass, and increased brain mass (Fig. 6a, b, Supplementary information, Fig. S5e). T2-weighted MRI analysis demonstrated enlarged lateral ventricles and enhanced signal intensity of the corpus callosum in *Prrc2a*^*f/f*^*; Olig*^*Cre+/-*^ mice (Fig. 6c). Similarly, black-gold staining and TEM analysis showed significant hypomyelination in the corpus callosum of *Prrc2a*^*f/f*^*; Olig2*^*Cre+/-*^ mice (Fig. 6d–g and Supplementary information, Fig. S5f). *Olig2*-Cre-mediated Prrc2a deletion significantly reduced proliferative capacity (Supplementary information, Fig. S5g and h). Pdgfrα^+^, CC1^+^/Olig2^+^ and Sox10^+^ cells were also significantly reduced in *Prrc2a*^*f/f*^*; Olig2*^*Cre+/-*^ mice (Fig. 6h, i, Supplementary information, Fig. S5i and j).

Accordingly, RNA-seq data showed oligodendroglial lineage specific genes were significantly downregulated (Fig. 6j and Supplementary information, Fig. S5k-m). Interestingly, most of the overlapped downregulated DEGs from *Nestin-*Cre- and *Olig2-*Cre- mediated Prrc2a-deficient mice were enriched in myelination and glial cell differentiation pathways (Fig. 6k and Supplementary information, Fig. S5n). The expression changes of oligodendroglial-specific genes were further confirmed by RT-qPCR analysis (Fig. 6l). To rule out the possible effect caused by *Olig2*-Cre-induced haploinsufficience of *Olig2* expression, we included *Prrc2a*^*f/+*^*; Olig2*^*Cre+/-*^ mice as a control and found that the expression levels of *Olig2* and myelin-related genes were significantly reduced in *Prrc2a*^*f/f*^*; Olig2*^*Cre+/-*^ mice (Supplementary information, Fig. S5o and p).

Consistent with these results, *Prrc2a*^*f/f*^*; Olig2*^*Cre+/-*^ mice were found to have a decreased lifespan and displayed significant motor defects (Supplementary information, Fig.S6a-c). Previous studies have demonstrated that Olig2 is required for oligodendrocyte and motor neuron specification in the spinal cord.^40^ We sought to ask whether the motor behavior defects were due to the reduced motor neuron number in *Prrc2a*^*f/f*^*, Olig2*^*Cre+/-*^ mice. Immunostaining analysis showed *Olig2*-*Cre* mice had low-recombination efficiency in motor neurons (ChAT-positive cells) and no significant difference of ventral motor neuron number in the spinal cord (Supplementary information, Fig. S6d and e). Furthermore, *Prrc2a*^*f/f*^*; Olig2*^*Cre+/-*^ mice also showed learning and memory defects (Supplementary information, Fig.S6f-k).

To rule out the role of astrocytes in hypomyelination, we next deleted Prrc2a in astrocytes by crossing *Prrc2a*^*f/f*^ mice with *Gfap*-*Cre* mice. Although *Gfap*-Cre significantly reduced the Prrc2a expression in astrocytes, no significantly developmental or behavioral abnormalities were observed in *Prrc2a*^*f/f*^*;Gfap*^*Cre+/-*^ mice (Supplementary information, Fig. S7a-f). Importantly, black-gold and immunostainings showed that *Prrc2a*^*f/f*^*;Gfap*^*Cre+/-*^ mice possessed normal myelination and oligodendrocyte populations (Supplementary information, Fig. S7g-j). Real-time qPCR and western blotting analysis also showed no significant change in the expression of oligodendrocyte marker genes (Supplementary information, Fig. S7k and l). Interestingly, while we observed no change in astrocyte number in *Prrc2a*^*f/f*^*;Gfap*^*Cre+/-*^ mice, we noticed that the expression levels of *Gfap* and *Aqp4* were significantly increased (Supplementary information, Fig. S7k-p).

### *Olig2* is an important target gene of Prrc2a

**Fig. 7:**
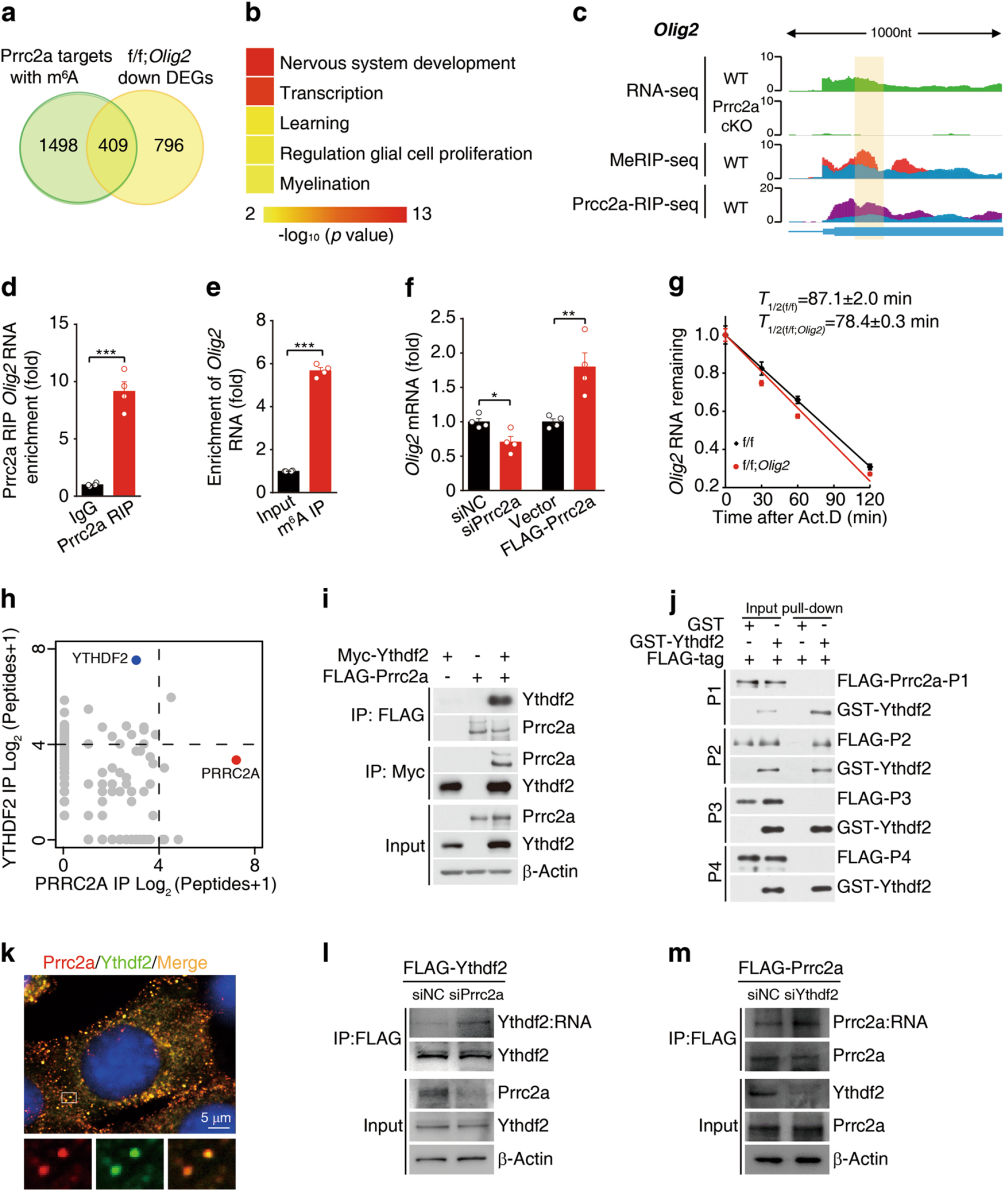
Prrc2a regulates *Olig2* mRNA stability in an m^6^A-dependent manner. **a** Overlap of genes with Prrc2a binding region containing m^6^A peaks and genes differentially expressed in *Prrc2a*^*f/f*^; *Olig2*^Cre+/-^ vs. control samples. **b** Representative Gene Ontology (GO) terms of the biological process categories enriched in differentially expressed transcripts with Prrc2a binding region containing m^6^A peaks. **c** Integrative Genomics Viewer (IGV) tracks displaying RNA-seq (upper panels), MeRIP-seq (Middle panel), and Prrc2a RIP-seq (bottom panel) read distributions in *Olig2* mRNA. Significant peaks are indicated with yellow highlight. **d** Prrc2a RIP-qPCR analysis of the region containing Prrc2a binding peak in *Olig2* mRNA from brain tissues of 4-week-old mice (two-tailed unpaired student’s *t*-test, ***P < 0.001, *n* = 4 per group). **e** Detection of m^6^A enrichment in *Olig2* mRNA from brain tissues of 4-week-old mice by m^6^A-RIP-qPCR (two-tailed unpaired student’s *t*-test, ***P < 0.001, *n* = 4 per group). **f** The mRNA level of *Olig2* in GL261 cell with or without Prrc2a knockdown/overexpression (two-tailed unpaired student’s *t*-test, *P < 0.05, **P < 0.01, *n* = 4 per group). **g** Cultured OPCs from control or *Prrc2a*^*f/f*^*; Olig2*^*Cre+/-*^ mice were exposed to actinomycin D (1 μg/ml), then RNA was isolated at indicated time points. RT-qPCR was performed to assess the half-lives of *Olig2* mRNA. The data were presented as means ± s.e.m. and the inserted numbers (*T*_1/2 (f/f)_ = 87.1 ± 2.0 min; *T*_1/2(f/f; *Olig2*)_ = 78.4 ± 0.3 min) showed the calculated half-times from four independent experiments. **h** Scatter plots of proteins bound to PRRC2A (red) vs. YTHDF2 (blue). (see also Supplementary information, Table S8). **i** Lysates from HEK293T cells transfected with indicated plasmids were subjected to immunoprecipitation with anti-FLAG or anti-Myc antibody, followed by immunoblotting with anti-Myc, anti-FLAG, or anti-β-actin antibody. **j** In vitro GST pull-down assay using purified GST-YTHDF2 and FLAG-Prrc2a-P1, FLAG-Prrc2a-P2, FLAG-Prrc2a-P3, or FLAG-Prrc2a-P4. **k** Confocal images of Prrc2a (red) and Ythdf2 (green) colocalization in HT-22 cells. **l** HT-22 cells stably expressing FLAG-Ythdf2 were transfected with Prrc2a siRNA or control siRNA. 72 h later, the cells were collected and subjected to PAR-CLIP analysis by immunoprecipitation with anti-FLAG antibody. The RNA products were labeled with biotin at 3′ end and then visualized by the chemiluminescent nucleic acid detection module. **m** HT-22 cells stably expressing FLAG-Prrc2a were transfected with Ythdf2 siRNA or control siRNA. 72 h later, the cells were collected and subjected to PAR-CLIP analysis by immunoprecipitation with anti-FLAG antibody. The RNA products were labeled with biotin at 3′ end and then visualized by the chemiluminescent nucleic acid detection module

To elucidate the molecular mechanism of Prrc2a in oligodendroglial specification, we compared the Prrc2a binding targets containing m^6^A peaks with downregulated DEGs from *Prrc2a* ^*f/f*^*; Olig2*^*Cre+/-*^ mice, and found that 409 DEGs were potentially regulated by Prrc2a via binding with m^6^A (Fig. 7a). GO analysis showed that they were enriched for functions related to nervous system development, transcription, and myelination (Fig. 7b). Further, we found that 24 oligodendroglial DEGs were also the targets of Prrc2a (Supplementary information, Fig. S8a and Supplementary information, Table S7). Among these potential target genes, we found *Olig2* expression was significantly reduced upon Prrc2a deficiency. Importantly, the m^6^A-RIP-seq and Prrc2a RIP-seq data revealed colocalization of m^6^A and Prrc2a binding peaks within the *Olig2* mRNA transcript (Fig. 7c). Previous studies have shown that Olig2 functions as an oligodendroglial lineage determination factor by controlling OPC specification, differentiation, and myelination.^40,41^ Therefore, we argue that *Olig2* might act as an important target of Prrc2a in regulating oligodendroglial specification. We further confirmed that Prrc2a directly binds the *Olig2* mRNA by using Prrc2a RIP-qPCR (Fig. 7d) and that the *Olig2* transcript bears an m^6^A modification by m^6^A-RIP-qPCR analysis (Fig. 7e).

To validate that *Olig2* mRNA is a Prrc2a target in cells, we performed Prrc2a knockdown and overexpression studies in GL261 cells. We found that Prrc2a knockdown reduced both mRNA and protein levels of Olig2, while Prrc2a overexpression increased Olig2 mRNA and protein levels (Fig. 7f and Supplementary information, Fig. S8b and c), suggesting that Prrc2a regulates *Olig2* mRNA and may affect its fate determination in cells. *Olig2* promoter luciferase assay showed that the overexpression/knockdown of Prrc2a had no effect on *Olig2* promoter activity (Supplementary information, Fig. S8d). These data indicate that Prrc2a alters *Olig2* expression through post-transcriptional regulation. We then mapped the region on *Olig2* mRNA that was regulated by Prrc2a and found that Prrc2a regulated *Olig2* expression through CDS region (Supplementary information, Fig. S8e-g), which was consistent with our MeRIP-seq and RIP-seq data (Fig. 7c). Combining our MeRIP-seq data with SRAMP software analysis,^42^ we identified a very high-confidence m^6^A site (GGA_112_CT, a conserved m^6^A methylation motif) in the *Olig2* CDS region (Supplementary information, Fig. S8h). Additionally, we found that overexpression/knockdown of Prrc2a had no effect on the mutant Olig2-CDS luciferase activity (Supplementary information, Fig. S8i and j). These data indicate that Prrc2a regulates *Olig2* expression through the m^6^A_112_ site. Consistently, the overexpression of Prrc2a stabilized the Olig2-Wildtype-CDS transcript but did not affect the Olig2-CDS A112T mRNA (Supplementary information, Fig. S8k). In addition, the Olig2 transcript displayed a significantly decreased half-life in Prrc2a deficient OPCs (Fig. 7g).

To explore the molecular mechanisms underlying the role of Prrc2a as a modulator of mRNA stability through the m^6^A modification, we screened for the interacting partners of PRRC2A by a tandem-affinity purification in HEK 293 T cells expressing FLAG-tagged PRRC2A (Fig. 7h). Notably, YTHDF2, a well-known m^6^A reader, was one of the potential PRRC2A interacting proteins. Interestingly, we also identified PRRC2A as one of YTHDF2-binding proteins in HEK293T cells (Fig. 7h). Further co-immunoprecipitation in HEK293T cells confirmed the interaction between PRRC2A and YTHDF2 (Fig. 7i). GST pull-down showed that the recombinant PRRC2A-P2 protein directly interacted with YTHDF2 (Fig. 7j and Supplementary information, Fig. S8l), and we observed that PRRC2A and YTHDF2 were colocalized in the granule-like subcellular organelles (Fig. 7k). Intriguingly, PRRC2A knockdown significantly increased the RNA-binding competence of YTHDF2 (Fig. 7l) and YTHDF2 knockdown augmented PRRC2A RNA-binding capacity (Fig. 7m), suggesting that YTHDF2 and PRRC2A compete for RNA binding.

### Fto-mediated internal m^6^A demethylation triggers hypomyelination

We next explored whether m^6^A demethylases is involved in regulating *Olig2* mRNA stability. We observed that the expression of *Olig2* was significantly increased upon knockdown of the RNA demethylase Fto (Supplementary information, Fig. S9a and b), while *Olig2* promoter activity was unchanged (Supplementary information, Fig. S9c). Similar to Prrc2a overexpression, *Fto* knockdown stabilized the *Olig2* CDS, but did not affect *Olig2* A112T mutant CDS (Supplementary information, Fig. S9d). In addition, the Olig2 transcript displayed a significantly increased half-life in Fto knockout OPCs (Supplementary information, Fig. S9e). These data suggest that Fto regulates *Olig2* mRNA in an m^6^A-dependent manner.

**Fig. 8:**
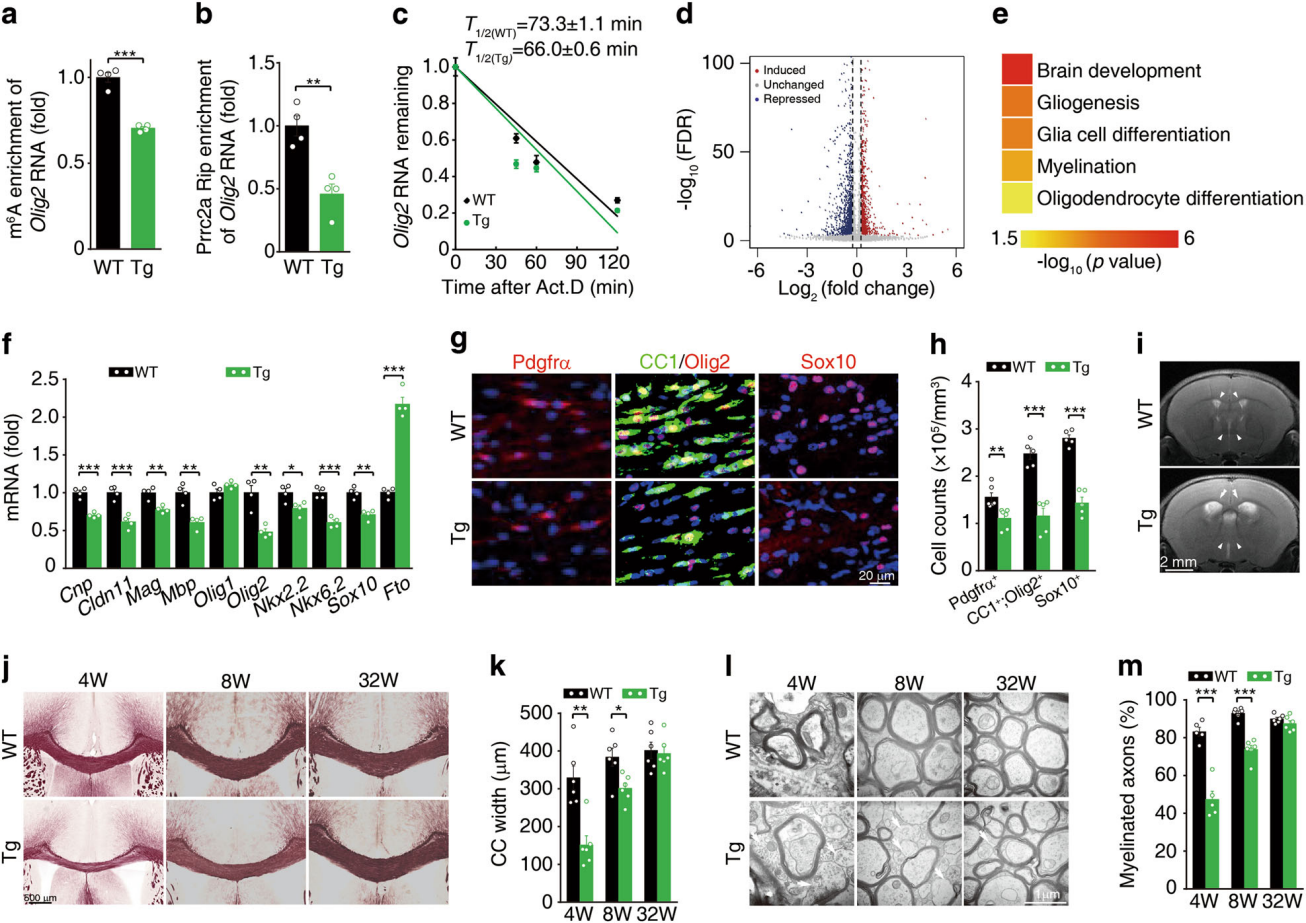
Fto transgene triggers hypomyelination. **a** MeRIP-qPCR analysis of *Olig2* RNA in m^6^A peak region in Fto transgenic (Tg) and wild-type (WT) mice at 4-weeks-old (two-tailed unpaired student’s *t*-test, ***P < 0.001, *n* = 4 per group). **b** Prrc2a RIP-qPCR analysis of *Olig2* RNA in m^6^A peak region in Fto transgenic (Tg) and WT mice at 4 weeks old (two-tailed unpaired student’s *t*-test, **P < 0.01, n = 4 per group). **c** Cultured OPCs from WT and Fto transgenic mice were exposed to actinomycin D (1 μg/ml), then RNA was isolated at indicated time points. RT-qPCR was performed to assess the half-lives of *Olig2* mRNA. The data were presented as means ± s.e.m. and the inserted numbers (*T*_1/2 (WT)_ = 73.3 ± 1.1 min; *T*_1/2(Tg)_ = 66.0 ± 0.6 min) show the calculated half-times from four independent experiments. **d** Volcano plot of RNA-seq data showing Fto-regulated genes from brain tissue samples of 4-week-old *Fto* transgenic vs. control mice. **e** Parts of GO terms of the biological process categories enriched in DEGs from *Fto* transgene vs. control samples. **f** Relative gene expression in hippocampus from 4-week-old Fto Tg and control mice (two-tailed unpaired student’s *t*-test, *P < 0.05, **P < 0.01, ***P < 0.001, *n* = 4 per group). **g** Immunostainings of Pdgfrα (P7), CC1/Olig2, or Sox10 (P28) in corpus callosum from mice with indicated genotypes. The quantification of Pdgfrα^+^, CC1^+^ Olig2^+^, and Sox10^+^ cells were shown in (**h**) (two-tailed unpaired student’s *t*-test, **P < 0.01, ***P < 0.001, Pdgfrα^+^: *n* = 6 each group; CC1^+^ Olig2^+^ cells: *n* = 5 per group, Sox10^+^ cells: *n* = 5 per group). **i** T2-weighted MRI of 4-week-old mice with indicated genotypes. **j** Black-gold staining of brain sections from 4-, 8-, and 32-week-old Fto Tg and control mice. **k** The quantification of corpus callosum width at the midline from 4-, 8-, and 32-week-old Fto Tg and control mice (two-tailed unpaired student’s *t*-test, *P < 0.05, **P < 0.01, 4w: *n* = 5 per group, 8w: *n* = 6 per group, 32w: *n* = 6 per group). **l** TEM analysis of the myelin fibers in the corpus callosum from mice with indicated genotypes and ages. The white arrowheads indicated the naked axons. **m** The percentage of myelinated axons in the corpus callosum from 4-, 8-, and 32-week-old mice with indicated genotypes (two-tailed unpaired student’s *t*-test, ***P < 0.001, 4w: *n* = 5 per group, 8w: *n* = 6 per group, 32w: *n* = 6 per group)

We then examined whether Fto regulates *Olig2* mRNA stability in vivo. In an Fto transgenic (Tg) mouse model, we observed a reduction in the m^6^A level of *Olig2* mRNA (Fig. 8a). Prrc2a-RIP-qPCR analysis showed that the Fto transgene reduced the capacity of Prrc2a to bind to *Olig2* mRNA (Fig. 8b). Additionally, Fto transgene significantly reduced the half-life of *Olig2* mRNA in cultured OPCs (Fig. 8c). RNA-seq data from brains of wild-type and *Fto* transgenic mice also showed that the Fto transgene had profound effects on gene expression (Fig. 8d). Interestingly, we found the DEGs (Fto transgenic *vs* wild type) were significantly enriched in glial cell differentiation and myelination pathways (Fig. 8e), among which nearly 25% DEGs were Prrc2a targets (Supplementary information, Fig. S9f and Supplementary information, Table S9). RT-qPCR analysis confirmed that *Fto* transgene reduced oligodendroglial lineage-specific gene expression (Fig. 8f and Supplementary information, Fig. S9g). The number of Pdgfrα^+^,  CC1^+^Olig2^+^ or Sox10^+^ cells was significantly reduced in *Fto* transgenic mice (Fig. 8g, h, Supplementary information, Fig. S9h and i). T2-weighted MRI analysis demonstrated enlarged lateral ventricles and enhanced signal intensity of the corpus callosum in *Fto* transgenic mice (Fig. 8i). Further black-gold staining and TEM analysis showed significant hypomyelination in brains from *Fto* transgenic mice at developmental stage (Fig. 8j–m and Supplementary information, Fig. S9j). Similarly, we found that *Fto* transgenic mice displayed significant locomotive and cognitive defects (Supplementary information, Fig. S9k-s). Collectively, Fto-mediated internal m^6^A demethylation triggers hypomyelination in mice.

## Discussion

In the present study, we demonstrated that Prrc2a, a novel m^6^A reader, plays a critical role in oligodendroglial specification and its loss of function leads to hypomyelination. Firstly, we identified Prrc2a as a reader of the m^6^A modification through a methylated RNA pull-down assay followed by mass-spectrometry in neural cells. The subsequent validation studies showed that Prrc2a prefers to bind m^6^A-contaning RNA. Functionally, mice with conditional knockout of Prrc2a with *Nestin*-Cre or *Olig2*-Cre displayed a shortened lifespan, impaired locomotion, and cognition. Further cellular analyses revealed that Prrc2a is involved in OPC proliferation and oligodendrocyte fate determination. Secondly, we found that Prrc2a post-transcriptionally regulates *Olig2* mRNA stability in an m^6^A-dependent manner. Finally, we found that Fto-mediated m^6^A demethylation promotes *Olig2* mRNA degradation and triggers hypomyelination in mice, which represents the first report of m^6^A as the substrate of Fto in mammals.

Here, we identified a novel m^6^A specific binding protein family, including Prrc2a and Prrc2c in neural cells by using methylated RNA bait. Despite the absence of a classical YTH domain, here we identified a new Prrc2a domain (named GRE domain) that could specifically bind methylated RNA, similar to the case of RNA-binding domain (RBD) of IGF2BPs that recognizes m^6^A-containing transcripts and regulates mRNA stability.^24^

Previous studies suggest that mRNA transcripts with m^6^A modifications tend to be less stable due to the relocation of such mRNAs to RNA decay sites by YTHDF2.^16,43^ However, some gene transcripts have been shown to be stabilized by the m^6^A modification,^13^ indicating there might be some unknown readers that could compete with YTHDF2 to dynamically regulate RNA stability. A recent study showed that IGF2BP family proteins stabilized m^6^A-containing transcripts through a distinct recognition pattern from YTHDF2.^24^ Here, we propose a possible model whereby PRRC2A interacts and competes with YTHDF2 for RNA binding.

Epidemiology studies demonstrated association between Prrc2a SNPs and the risk of cancer,^44,45^ rheumatoid arthritis,^46^ insulin-dependent diabetes mellitus (IDDM)^47^, and obesity.^48^ However, the roles of Prrc2a in the nervous system remained unknown despite its high expression in the central nervous system.^49^ Our present work indicates that Prrc2a is a new class of m^6^A reader that controls neural development. Despite no significant neurogenesis abnormalities, significant hypomyelination in the corpus callosum from Prrc2a conditional knockout mice was observed. Therefore, we argue that increased brain mass might be due to enlarged lateral ventricles and/or hypomyelination-induced brain edema. Although recent reports reveal that m^6^A plays important roles in regulating neurogenesis, neural stem cell self-renewal, axon regeneration, and local translation,^30–32,50^ our study is the first to delineate the role of m^6^A in regulating oligodendroglial specification and myelination mediated by a new m^6^A reader Prrc2a.

Prrc2a deficiency reduced the proliferation of OPCs both in vivo and in vitro, but we observed that Prrc2a deficiency promoted oligodendrocyte differentiation in vitro. In addition, myelin-related transcripts were significantly increased in Prrc2a deficient oligodendrocytes in vitro (Fig. 5l). However, Prrc2a deletion significantly reduced myelin-related transcript and led to hypomyelination phenotype in vivo (Figs 2f–l and 4e, Supplementary information, Fig. S3c). The paradoxical myelin-related gene expressions might be due to the reduced OPCs numbers in vivo and different differentiation efficiency of OPCs between in vivo and in vitro conditions.

Recent reports have shown that microRNAs and chromatin modifications play important roles in regulating oligodendrocyte differentiation and myelination.^51,52^ However, the post-transcriptional regulation during this process was completely unknown. Our current discovery uncovers a new role for mRNA modifications in the process of oligodendroglial specification and highlights the importance of post-transcriptional regulation in neural development and CNS disorders.

In summary, our study found that Prrc2a deficiency impaired oligodendroglial specification and induced hypomyelination through regulating *Olig2* mRNA processing (working model, Supplementary information, Fig. S10). Our study is the first to link m^6^A RNA modifications with the process of myelination, and suggests a new direction for the development of effective therapeutic strategies for myelination-related disease processes.

## MATERIALS AND METHODS

### Animals

*Prrc2a*^*f/f*^ mice (generated by Dr. Fengchao Wang, National Institute of Biological Science, Beijing) were crossed with *Nestin*-*Cre* transgenic mice. The resulting *Prrc2a*^*f/+*^;*Nestin*^*Cre+/-*^ mice were then crossed with *Prrc2a*^*f/f*^ mice to obtain *Prrc2a*^*f/f*^;*Nestin*^*Cre+/-*^ study subjects and their control littermates (*Prrc2a*^*f/f*^ mice and *Prrc2a*^*f/+*^;*Nestin*^*Cre+/-*^ mice). *Prrc2a*^*f/+*^;*Nestin*^*Cre+/-*^ littermates were included as controls to rule out nonspecific effects of the *Nestin-Cre* transgene.^53^ Similarly, specific deletion of Prrc2a in astrocytes was obtained through crossing *Prrc2a*^*f/f*^ mice with *Gfap*-*Cre* transgenic mice (Jackson Laboratory, Stock Number: 024098, Cre recombinase was driven by the mouse glial fibrillary acidic protein promoter). The offsprings *Prrc2a*^*f/f*^*; Gfap*^Cre+/-^, and *Prrc2a*^*f/f*^ were used. In parallel, to knockout Prrc2a in oligodendroglial lineage, we crossed *Prrc2a*^*f/f*^ mice with *Olig2-Cre* transgenic mice^54^ (provided by Dr. Bo Xiao, West China Hospital, Sichuan University; Jackson Laboratory, Stock Number: 011103). The offsprings *Prrc2a*^*f/f*^*; Olig2*^Cre+/-^, and *Prrc2a*^*f/f*^ were used. Fto transgenic mice and Fto knockout mice were kind gifts from Dr. Pumin Zhang (National Center for Protein Sciences, the PHOENIX Center, Beijing). Fto transgenic mice were generated using the lentiviral method. Briefly, the mouse Fto (mFto) coding sequence was cloned into the lentiviral construct and the expression was driven under ubiquitin C promoter. The floxed *Prrc2a* gene was identified *via* PCR using forward primer 5′-GCAACTGAAGAAACGGTGGA-3′ and reverse primer 5′-AAGGCAAC TAACAGACCAGATGAA-3′, yielding PCR products of 413 and 533 bp for the WT and floxed alleles, respectively. For *Nestin*-Cre, *Gfap*-Cre and *Olig2*-Cre, the following common primers were used: Cre-forward primer 5′-GATCTCCGGTATTGAAACTCCAGC-3′and Cre-reverse primer 5′-GCTAAACATGCTTCATCGTCGG-3′, and the PCR yielded a 646 bp product. Genotyping of Fto transgenic mice using the Fto-Tg-forward primer 5′-GAGGGGAGGGATAAGTGAGG-3′ and Fto-Tg-reverse primer 5′-CATCTTTGGGGGTCAGGTAA-3′ yielded a 426 bp product. Fto-KO-forward primer 1# 5′-CAGTGGTCTGAGGACAAGCA-3′, Fto-KO-forward primer 2# 5′-TGGATCCGTGCATCTGTAAA-3′ and Fto-KO-reverse primer 5′-CGACAATCGAGATGGTGATG-3′ were used for genotyping of Fto knockout mice. All mice were in the C57BL/6 J background.

Mice were maintained under conditions of a 12 h light/dark cycle at 23 °C and were provided with food and water ad libitum in the Animal Care Facility at the Institute of Basic Medical Sciences (Beijing, China). All animals’ experiments were approved by and conformed to the guidelines of the institutional animal care and use committee at the Institute of Basic Medical Sciences (Beijing, China).

### Motor coordination test

Motor performance was estimated using an accelerating Rota-Rod (LE8200, Panlab, Harvard Apparatus) as previously described.^55^ Briefly, mice were trained on the Rota-Rod at 10 rpm. three times per day (at 1 h intervals) for 2 days before testing. During testing, the rod was accelerated from 4 to 40 rpm over a period of 300 s. Each result represents the average endurance of three consecutive measurements performed at 1 h intervals.

### Grip strength test

Grip strength test was estimated using a grip strength meter (BSBIOGS3, Panlab, Harvard Apparatus). During the test, the grip strength meter was positioned horizontally and the mice were held by the tail and lowered towards the apparatus. The animals were allowed to grasp the metal grid and then pulled backwards in the horizontal plane. The force applied to the grid just before the loss of grip is recorded as the peak tension. The forces were measured in Newton.

### Morris water maze test

The water maze task was performed as previously described^56^ with some modifications. It consisted of three phases: 1) 4 days with a visible platform (2 trials/day); 2) 5–6 days with a hidden platform (two trials/day); 3) Probe trials, during which the platform was removed from the maze, lasted 1 min and were performed to assess the retention of previously acquired information. Probe was conducted 24 h later after the last trail of the whole learning process. Mice were tracked by a video camera (Sony) in both trails and probe. Collected data were analyzed by SMART 2.5 software (Panlab, Harvard Apparatus). Statistical analyses used two-way ANOVA followed by Bonferroni test.

### Callosal compound action potential (CAP) recording

#### Slice preparation

Eight-week-old mice were anesthetized with intraperitoneal injection of sodium pentobarbital (30 mg/kg) and then decapitated. The brains were dissected out and transferred to an ice-cold slicing solution (2.5 mM KCl, 1.25 mM NaH_2_PO_4_, 26 mM NaHCO_3_, 10 mM Dextrose, 213 mM Sucrose, 2 mM MgSO_4_, 2 mM CaCl_2_), which was bubbled with mixed gas (95% O_2_, 5% CO_2_). Coronal slices were cut (400 µm thick, plates 34–38, Paxinos and Franklin mouse brain atlas) from brain regions containing the corpus callosum using a vibratome (Leica VT1200S). The slices were then transferred to an incubation chamber filled with Artificial cerebrospinal fluid (ACSF) (126 mM NaCl, 2.5 mM KCl, 1.25 mM NaH_2_PO_4_, 26 mM NaHCO_3_, 25 mM Dextrose, 2 mM MgSO_4_, 2 mM CaCl_2_; 315–325 mOsm, pH = 7.2–7.3) and maintained at 34.5 °C for 1 h. After incubation, slices were kept in the same solution at room temperature and allowed to equilibrate at least 30 min prior to recording.

#### Electrophysiological recordings

Slices were transferred to a recording chamber perfused at 2 ml/min rate with aerated ACSF at 21.5 °C. As described in a previous report,^57^ a tungsten bipolar electrode was used for stimulation in the corpus callosum (CC) of one hemisphere and a glass electrode (impedance of 1–3 MΩ) filled with ACSF is placed in the contralateral hemisphere for recording. The stimulating pulses of 0.1 ms duration and 4 mA current were applied via an isolator (ISO-Flex, A.M.P.I). Evoked CAPs were recorded by a Multiclamp 700B amplifier (Molecular Devices) and sampled by a Power3 1401 (CED, Cambridge Electronic Design) at 25 kHz using Spike2 software (Bessel filter set to 10 KHz) for offline analysis. For each slice, we recorded two trials with the same stimulation site and different recording sites.

#### Data analysis and statistics

For each trial, 20–60 repeat responses were averaged for waveform analysis. Conduction velocity was estimated by the difference of two different distances between the stimulating and recording electrodes divided by the difference of corresponding peak latency in the same slice, that is, Fast velocity = δDistance/δT1; Slow velocity = δDistance/δT1. CAP amplitude can be measured as the vertical distance from the local negative peak of two depolarizing phases of the CAPs (Amp.1 and Amp.2) to a tangent joining preceding and following positivity.^57^

### Histological analysis

Brain tissue was dissected and fixed for three days in 4% paraformaldehyde, embedded in paraffin blocks and sectioned. Tissue sections were stained with hematoxylin/eosin following standard procedures.

### Immunohistochemistry

The tissues were post-fixed using 4% paraformaldehyde and dehydrated with gradient sucrose (10%, 20%, and 30%) in PBS. Coronal sections (40 μm in thickness) were sliced with Leica CM3050S and processed for immunohistochemistry. In detail, the brain slices were immersed in PBS twice and incubated in blocking buffer (PBS containing 0.4% Triton X-100, 2% horse serum, and 1% BSA) for 1 h at room temperature. For astrocytes labeling, the slices were treated with a 1:1000 dilution of mouse monoclonal anti-GFAP (MAB360, Millipore) or 1:400 dilution of rabbit polyclonal anti-ALDH1L1 (Ab87117, Abcam, Cambridge, UK) followed by biotinylated goat anti-mouse/rabbit IgG and streptavidin-conjugated HRP (Vectastain ABC kit, Vector Laboratories, Burlingame, CA, USA) and positive immunostaining was visualized using 3,3′-diaminobenzidine (DAB) followed by a reaction with hydrogen peroxide (DAB kit, Vector Laboratories). Selectively, some sections were incubated with anti-GFAP (1:1000, Z0334, Dako, Santa Clara, CA,USA), anti-Olig2 (1:500, AB9610, Millipore), anti-CC1 (1:500, OP80, Millipore), anti-ChAT (1:300, AB144P, Millipore), anti-ChAT (1:300, Sc-55557 Santa Cruz, Dallas, TX, USA), anti-Sox10 (1:500, Sc-365692, Santa Cruz), anti-BrdU (1:1000, 66241-1-Ig, Proteintech Group), anti-Ki67 (1:500, ab15580, abcam), anti-Pdgfrα (1:400, 558774, BD Bioscience), anti-NG2 (1:200, 05–710, Millipore), and anti-Prrc2a (1:200, Sc-78859, Santa Cruz) overnight and followed by fluorescence-conjugated secondary antibodies (Jackson Immunoresearch, Pennsylvania, USA) for 1 h at room temperature. Stained sections were mounted onto slides and images were acquired using a confocal microscope (Leica). For Pdgfrα, NG2 and Prrc2a immunostaining, tissues were fixed using 2% paraformaldehyde for no more than 24 h. Specially, for Prrc2a immunostaining, tissues were subjected to antigen retrieval in EDTA-Tris buffer (pH9.0, ZLI-9068, ZSGB-Bio, Beijing, China).

### BrdU labeling

For OPC and astrocytes proliferation analysis in vivo, we injected mice intraperitoneally with BrdU (50 mg/kg bodyweight, HY-15910, MedChemExpress) at P6. Two hours later, the perfused brains were dissected and fixed in 2% paraformaldehyde for 24 h. Brains were dehydrated with gradient sucrose (10%, 20% and 30%), embedded in OCT compound and sectioned coronally (40 μm-thickness) on a Leica CM3050S cryostat. Brain sections were incubated with 1 M HCl for 10 min on ice, 2 M HCl for 10 min at room temperature, and 20 min at 37 °C, then neurtralized with 0.1 M borate buffer for 10 min at room temperature, continuing with the standard staining procedure as described in the immunochemistry.

### Black-Gold Staining

Black-gold staining was performed using Black-Gold II myelin staining kit (AG105, Millipore) according to manufacturer’s instructions. Briefly, 0.3% Black-Gold II and 1% sodium thiosulfate solutions were pre-heated to 60 °C. Brain sections were immersed in water to rehydrate for 5 mins. Then pre-warmed Black-Gold II solution was added and the incubation lasted for 12 min at 60 °C. During staining, the slides were monitored at 2–3 min intervals to determine the extent of labeling. When the finest myelinated fibers were stained to dark red to black, we stopped staining. Then slices were incubated for 3 min at 60 °C with sodium thiosulfate solution (1%). Stained slices were mounted on slides after water washing twice.

### Magnetic resonance imaging (MRI) analysis

We carried T2-weighted Magnetic Resonance Imaging in vivo at 7.0 T using Bruker BioSpin MRI GmbH system according to standard procedure.

### Transmission electron microscopy (TEM) analysis

For TEM, mice were perfused sequentially with normal salting and 2% glutaraldehyde/2% paraformaldehyde in 0.1 M cacodylate buffer. After perfusion, corpus callosum was dissected and post-fixed overnight in the same fixative buffer. Tissues were then fixed in OsO4 for 1 h and embedded in epoxy resin. Ultra-thin sections were obtained using Ultracut UCT (Leica) and stained with 2% uranyl acetate and lead citrate. Electron micrographs were imaged in Phillips Tecnai 10 transmission electron microscope (Hillsboro, Oregon, USA) using FEI software.

### Primary oligodendrocyte precursor cell culture

The procedure was modified from a previous report.^38^ Briefly, cerebral cortexes were dissected from E14.5-17.5 pregnant mice and then dissociated by mechanical trituration until the cell suspension has no small clumps. The suspension was passed through 70 μm Nylon cell strainer (FALCON) to obtain single cell suspension. We counted the cell number and added 6× 10^4^ cells per well in Ultra-Low Attachment Surface six well plate (Corning). The cells were growth in neurosphere growth medium (DMEM/F12 plus 25 μg/ml insulin, 50 μg/ml apo-transferrin, 20 nM progesterone, 60 μM putrescine, 30 nM sodium selenite, 20 ng/ml EGF, 20 ng/ml bFGF, and 10% methylcellulose) for about 4 days until the neurosphere size around 100–200 μm. We gradually changed the neurosphere growth medium to B104 conditional medium (30% B104 medium [the supernatant of B104 cell with DMEM/F12 + 1 × N2 supplement cultured for 4 days] and 70% neural culture medium [DMEM/F12 plus 25 μg/ml insulin, 100 μg/ml apo-transferrin, 20 nM progesterone, 60 μM putrescine, and 30 nM sodium selenite]) by replacing one-fourth of the former medium with the latter medium every other day for 14 days. Then oligospheres were dissociated by mechanical trituration followed by passing through a 70 μm Nylon cell strainer. The single cell suspensions were plated in the poly-ornithine-coated plate for OPC proliferation in the OPC medium (DMEM plus 4 mM L-glutamine, 1 mM sodium pyruvate, 0.1%BSA, 50 μg/ml apo-transferrin, 30 nM sodium selenite, 10 nM D-botin, 10 nM hydrocortisone, 10 ng/ml PDGF-AA, and 10 ng/ml bFGF) or for differentiation in differentiation medium (DMEM plus 4mM L-glutamine, 1 mM sodium pyruvate, 0.1%BSA, 50 μg/ml apo-transferrin, 30 nM sodium selenite, 10 nM D-botin, 10 nM hydrocortisone, 15 nM T3, 10 ng/ml CNTF, and 5 μg/ml NAC).

### Immunoblotting

Immunoblotting was performed as previously described.^55^ Briefly, tissues were lysed in lysis buffer and protein concentration was determined by Bradford. Proteins were separated on polyacrylamide gel and transferred to PVDF membrane. The membrane was blocked in 5% milk and subsequently probed with primary antibodies overnight at 4 °C, then incubated with HRP-conjugated secondary antibodies for protein detection. The antibodies used were anti-GFAP (Z0334, Dako, Santa Clara, CA,USA), anti-ALDH1L1 (Ab87117, Abcam, Cambridge, UK), anti-MBP (78896, Cell Signaling Technology, Cambridge, MA, USA), anti-Olig2 (AB9610, Millipore, Billerica, MA, USA), anti-Prrc2a (Sc-78859, Santa Cruz, Dallas, TX, USA), anti-FLAG (F1804, Sigma), anti-Myc (M047-3, MBL, Woburn, MA,USA), anti-Ythdf1 (17479-1-AP, Proteintech Group, Campbell Park, Chicago, IL,USA), anti-Ythdf2 (24744-1-AP, Proteintech Group), anti-PARP1 (ab191217, Abcam), anti-β-actin (60008-1-Ig, Proteintech Group), anti-GAPDH (CW0266A, CWBiotech, Beijing, China), and anti-β-tubulin (CW0098A, CWBiotech). Polyclonal rabbit anti-FTO antibody was affinity-purified from rabbits immunized with 6 × His tagged full-length human FTO protein as previously reported.^5^ Polyclonal rabbit anti-ALKBH5 antibody was generated against synthesized peptide by CWBio (Beijing) as previously reported.^4^

### Real-time quantitative PCR

Total RNA from tissues was extracted using TRIZOL (Invitrogen). Reverse transcription was performed using random primers. Quantitative real time-PCR was performed using Trans Start Green qPCR Super Mix (Transgene Biotechnology, Beijing, China) in a Stratagene Mx3005P (Agilent Technologies). *β-actin* was used as a housekeeping gene for input normalization. The mRNA expression was measured by quantitative PCR using the Delta-Delta CT method. Primers for quantitative PCR were shown in Supplementary information, Table S10.

### Plasmid constructs and stable expression cell selection

DNA fragments corresponding to full-length Prrc2a (isoform 1), p1 (residue 1–760), p2 (residue 761–1408), p3 (residue 1409–1720), and p4 (residue 1720–2158) were amplified from a mouse cDNA library by PCR and inserted into p3 × FLAG-CMV-10 Expression Vector (Sigma-Aldrich, St Louis, MO, USA) using the HindIII and BamHI restriction sites. N-terminal 3 × FLAG- and C-terminal HA-tagged mouse full-length Prrc2a was inserted into retroviral vector PQCXIH using the NotI and BamHI restriction sites. To generate Prrc2a stable overexpression cells, HT-22 and GL261 cells were infected with the retroviral vector PQCXIH encoding 3 × FLAG-Prrc2a-HA and selected with hygromycin. In our study, HT-22 is a mouse hippocampal cell line and GL261 is a mouse glioma-derived cell line which has been reported to express OPC marker genes.

### Plasmid transfection and RNA interference

Mouse Prrc2a, Fto, and Alkbh5 siRNAs were designed and synthesized by Genepharma Corporation (Suzhou, China). The following siRNA were synthesized and used in the study: Prrc2a siRNA 1#: 5′-CAUGAAGAGGUUGACUAUA-3′, Prrc2a siRNA 2#: 5′-GCUUGUAUAUAGAUUAUAA-3′, FTO siRNA: 5′-GCAGCUGAAAUACCCUAAA-3′, ALKBH5 siRNA: 5′-ACAAGUACUUCUUCGGCGA-3′, Scrambled siRNA (siNC): 5′-UUCUCCGAACGUGUCACGU-3′. Transfections were performed with Lipofectamine RNAiMAX (Invitrogen) for siRNA, and Lipofectamine 2000 (Invitrogen) for plasmid following the manufacturer’s instructions.

### Luciferase assay

DNA fragments corresponding to Olig2 promoter (−1680-0) were amplified from mouse genomic DNA by PCR and inserted into pGL3-Basic Vector (Promega, Madison, WI, USA) by using the KpnI and HindIII restriction sites. Olig2 5′UTR, CDS and 3′UTR were amplified from a mouse brain cDNA library by PCR and inserted into pGL3-Promoter Vector (Promega) using the HindIII, NcoI or XbaI restriction sites. The Olig2-CDS-A112T mutant was generated by site-directed mutagenesis. All plasmids and mutations were verified by sequencing. 0.05 μg Luciferase reporter plasmid and 0.05 μg TK-Renilla were co-transfected into GL261 cells in a 24-well plate using lipofectamine 2000 (Invitrogen). After 24 h, cell extracts were obtained and firefly/Renilla luciferase activities were measured using a Promega Dual-Luciferase reporter system.

### mRNA half-life measurement

DNA fragments corresponding to Olig2 CDS were amplified from a mouse brain cDNA library by PCR and inserted into p3 × FLAG-CMV-10 Expression Vector (Sigma) by using the HindIII and EcoRI restriction sites.

To measure the half-life of Olig2 CDS, GL261 cells were transfected with FLAG-tagged Olig2-CDS or mutants together with FLAG-Prrc2a, Fto siRNA or their controls. After 24 h (overexpression) or 48 h (RNAi), actinomycin D (2 μg/ml, HY-17559, Medchemexpress, Monmouth Junction, NJ, USA) was added into the cell culture medium. Total RNA was prepared and subjected to RT-qPCR analysis using Olig2 primer #2 (Supplementary information, Table S10).

### Protein purification in mammalian cells

HEK293T cells were transiently transfected with FLAG-tagged Prrc2a P1-4 plasmids, after 48 h, cells were lysed with lysis buffer (50 mM Tris-HCl, pH 7.4, 500 mM NaCl, 1% NP-40) and then sonicated (10% output, 10 s on, 20 s off) for 1 min. Cell debris was removed by centrifugation and the crude lysates were incubated with FLAG beads for 4 h at 4 °C. After five times washing with lysis buffer, the beads-bound proteins were eluted with 1 mg/ml 3 × FLAG peptide for 1 h at 4 °C. The quality of proteins was tested by coomassie brilliant blue staining.

### Protein purification in *E. coli*

The human YTHDF2 gene was subcloned into pGEX-5 × -2 expression vector with GST-tag. Then recombinant GST-YTHDF2 protein was induced into *E.coli* strain BL21 (DE3) and purified by FPLC using Bio-Scale Mini Profinity GST cartridge (Bio-rad) according to the manufacturer’s instructions. The quality of proteins was tested by coomassie brilliant blue staining.

### Expression and purification of recombinant protein in insect cell

Recombinant baculovirus expressing Prrc2a-p2 (residue 761–1408) carrying an N-terminal hexahistidine (6 × His) and C-terminal FALG tag was generated by Bac to Bac system (Invitrogen). Briefly, His_6_-Prrc2a-p2-FLAG DNA fragment was cloned into PH7 donor vector (modified from pFastBac I) by LIC. The PH7-His_6_-Prrc2a-p2-FLAG plasmid was transformed into DH10 competent cell to generate recombinant bacmid virus DNA. Then the recombinant Bacmid DNA was transfected into Sf9 insect cells and incubated at 27 °C to generate recombinant baculovirus. We isolated P1 baculoviral stock 72 h post transfection and amplified baculoviral stock. The high-titer P3 baculoviral stock was used to infect Hi5 cells for expressing recombinant protein. After suspension cultured for 72 h, cells were harvested and resuspended in lysis buffer (20 mM Tris 8.0, 300 mM NaCl, 10 mM imidazole, 1 × protease inhibitors). The recombinant protein was enriched by NTA-Ni and eluted using elution buffer (20 mM Tris 8.0, 300 mM NaCl, 400 mM imidazole). The eluted recombinant protein was exchanged to low salting buffer (10 mM Tris, pH 8.0, 150 mM NaCl) by superdex 200. Then protein was concentrated and frozen at −80℃.

### Isolation of cytoplasmic fractions

Cultured cells were trypsinized and washed once with cold PBS, and then incubated with 5 volumes of buffer A (10 mM HEPES pH 7.9, 1.5 mM MgCl2, 10 mM KCl, 0.5 mM DTT, 1 × Protease Inhibitor Cocktail) for 10 min on ice. The cells were centrifuged at 2000 × rpm for 10 min at 4 °C. The pellets were resuspended in 2 volumes of buffer A and slowly forced through the 1 ml syringe needle for 10 strokes to ensure complete cell lysis. The homogenate was centrifuged at 2000 × rpm for 10 min at 4 °C and the supernatant was mixed with 0.11 volume of buffer B (0.3 M HEPES pH 7.9, 1.4 M KCl and 0.03 M MgCl2), and centrifuged at 10,000 × g for 60 min at 4 °C. The supernatant from this step was designated as the cytoplasmic fraction. The pellet collected from the 2000 rpm centrifugation was subjected to a second centrifugation at 25,000 × g for 20 min at 4 °C to remove cytoplasmic residuals. The pellets were then resuspended in 2 volumes of buffer C (20 mM HEPES pH 7.9, 25% (v/v) glycerol, 0.42 M NaCl, 1.5 mM MgCl2, 0.2 mM EDTA, 0.5 mM phenylmethylsulfonyl fluoride, and 0.5 mM DTT). The suspension was vigorously forced through the 1 ml syringe needle for 10 strokes for complete lysis of nuclei, and then centrifuged at 25,000 × g for 30 min at 4 °C. The supernatant was designated as the nuclear fraction. The nuclear and cytoplasmic fractions were analyzed by western blotting using PARP1 and β-Tubulin as nuclear and cytoplasmic markers, respectively.

### RNA affinity chromatography and mass spectrometry identification

The biotin-labeled RNA oligonucleotides without (Oligo-A) or with m^6^A (Oligo-m^6^A): 5′-biotin-AGAAAAGACAACCAACGAGGGXCUCAUCAU-3′(X = A or m^6^A), were synthesized by the Chemical Synthesis Center of the National Institute of Biological Sciences, Beijing. In vivo RNA pull-down assays were carried out using HT22 cell cytoplasmic extracts. Briefly, extracts were pre-cleared for 1 h at 4 °C by incubation with streptavidin-conjugated magnetic beads (NEB) in binding buffer (50 mM Tris-HCl pH 7.5, 250 mM NaCl, 0.4 mM EDTA, 0.1% NP-40, 1 mM DTT, 0.4 U/μl RNasin). Biotin-labeled RNA oligonucleotides were incubated with pre-cleared cytoplasmic extracts for 2 h at 4 °C under gentle rotation together with streptavidin-conjugated magnetic beads which were pre-cleared by incubation with 0.2 mg/ml tRNA (Sigma) and 0.2 mg/ml BSA (Amresco) for 1 h at 4 °C under gentle rotation. Beads were washed three times with wash buffer (50 mM Tris-HCl pH 7.5, 250 mM NaCl, 0.4 mM EDTA, 0.1% NP-40, 1 mM DTT, 0.4 U/μl RNasin). Samples were subjected to SDS-PAGE and visualized by coomassie blue staining. The protein-containing gel slices were washed twice with MS-grade water, and then successively destained with acetonitrile. Proteins were reduced with 10 mM DTT in 25 mM ammonium bicarbonate at 56 °C for 1 h and alkylated by 55 mM iodoacetamide in 25 mM ammonium bicarbonate in the dark at room temperature for 45 min. Finally, gel pieces were thoroughly washed with 25 mM ammonium bicarbonate in water–acetonitrile (1:1, *v/v*) solution and completely dried in a SpeedVac. Proteins were incubated for 30 min in 20 μl of trypsin solution (10 ng/ml in 25 mM ammonium bicarbonate) on ice before 25 μl of 25 mM ammonium bicarbonate was added and the mixture was incubated at 37 °C overnight. The digestion reaction was stopped by addition of 5% formic acid (FA) that made pH < 4; the digestion mixture was briefly spun down and the supernatant containing the peptides were analyzed via LC-MS using a nanoLC-LTQ-Orbitrap XL (Thermo Fisher Scientific, San Jose, CA). Peptide samples were loaded onto columns 3 cm in length and 150 μm in inner diameter which were packed in house with ReproSil-Pur C18-AQ 5 μm particles (Dr. Maisch GmbH, Ammerbuch). The high performance liquid chromatography (HPLC) columns 15 cm in length and 75 μm in inner diameter were packed in house with ReproSil-Pur C18-AQ 3 μm particles (Dr. Maisch GmbH, Ammerbuch). Peptide mixtures were separated using linear gradients of 90 min and a two buffer system: buffer A (0.5% FA/H_2_O) and buffer B (0.5% FA/ACN). The flow rate was set to 300 nl/min. Peptides eluting from the column were directly sprayed into the mass spectrometer with a spray voltage of 2.1 kV and a capillary temperature of 225 °C. The quadrupole linear ion trap (LTQ) mass spectrometer was operated in data-dependent mode with the initial MS scan ranging from 300 to 1600 Da. The 10 most-abundant ions were automatically selected for subsequent collision-activated dissociation. To minimize peptide re-sequencing, dynamic exclusion was enabled within a time window of 90 s. Raw MS files were processed using Proteome Discoverer (Version 1.4.0.288, Thermo Fisher Scientific) with SEQUEST as the search engine. MS/MS spectra were searched against the UniprotKB human database and supplemented with known contaminants. Cysteine carbamidomethylation was set as a fixed modification, and N-terminal acetylation and methionine oxidation were set as variable modifications. Peptide mass and fragment mass tolerances were set at 20 ppm and 0.6 Da, respectively, and a maximum of two missed cleavage sites were allowed. Peptide identifications were filtered at a 1% false discovery rate.

### Photoactivatable ribonucleotide crosslinking and immunoprecipitation (PAR-CLIP)

HEK 293 T cells were transfected with 3 × FLAG-tagged Prrc2a p1, p2, p3, and p4 plasmids or HT-22 cells stably expressing FLAG-Ythdf2 or FLAG-Prrc2a were transfected with siRNA. Cells were cultured in medium supplemented with 4-SU (200 μM) for 16 h (Sigma), irradiated with 365 nm UV light for induction of crosslinking. Immuno-precipitated protein-RNA complexes were subjected to PAR-CLIP-biotin chemiluminescent nucleic acid detection. The protein-RNA complex was labeled with biotin using the RNA 3′End Biotinylation kit (Thermo) following the manufacturer’s instructions. After washing three times with IP wash buffer, beads were resuspended in 20 μl 2 × LDS loading buffer (Invitrogen) and 40 μl 1 × LDS loading buffer (Invitrogen), boiled at 95 °C for 10 min. To detect RNA-protein complexes, the samples were separated by SDS-PAGE and visualized by the chemiluminescent nucleic acid detection module (Thermo) following the manufacturer’s instructions.

### *In vitro RNA* pull-down and HPLC analysis

1 μg purified mRNAs were fragmented into ~100 nt pieces (save 0.2 μg from the same sample as input) and then incubated with 6 × His-Prrc2a-P2-FLAG protein with a final concentration of 500 nM in 300 µl IPP buffer (150 mM NaCl, 0.1% NP-40, 10 mM Tris, pH 7.4, 40 U/ml RNase inhibitor, 0.5 mM DTT), and the solution was mixed with anti-FLAG M2 magnetic beads (Sigma) for 2 h at 4 °C with rotation. The beads were washed four times with 500 µl IPP buffer each time. 0.4 ml TRIzol reagent was added to the beads and further purified was performed according to the manufacturer’s instructions. The purified fraction was dissolved in 15 µl water, and saved as Prrc2a-P2-bound. LC-MS/MS was used to measure the level of m^6^A in each sample of input and Prrc2a-P2-bound.

### EMSA

Purified 6 × His-FLAG-tagged wild-type Prrc2a-P2 protein was diluted to a series of concentrations of 0.05 μM, 0.1 μM, 0.2 μM, and 0.4 μM in binding buffer (50 mM Tris-HCl pH 7.5, 100 mM NaCl, 0.4 mM EDTA, 0.1% NP-40, and 40 U ml^–1^ RNasin, 1 mM DTT, 50% glycerol, 5 ng/μl BSA). 1 μl synthesized RNA probe with or without m^6^A (300 nM final concentration) and 1 μl purified protein (5 nM, 10 nM, 20 nM, and 40 nM final concentration, respectively) were mixed and incubated at room temperature for 30 min. Then, 1 μl glutaraldehyde (0.2% final concentration) was added into the mixture and incubated at room temperature for 15 min. The entire RNA-protein mixture was mixed with 5 μl 5 × Hi-Density TBE Sample buffer and separated on 6% TBE gel on ice for 30 min at 80 V. The gel was transferred onto positive charged nylon transfer membrane (GE Healthcare) and nucleic acids were detected by the chemiluminescent nucleic acid detection module (Thermo) following the manufacturer’s instructions. Quantification of each band was carried out using Quantity One software (Bio-Rad). The K_d_ (dissociation constant) was calculated with nonlinear curve fitting (Function Hyperbl) of Origin 8 software with y = *P*_1_ × *X*/(*P*_2_ + *X*), where y is the ratio of(RNA-protein)/[(free RNA) + (RNA-protein)], *X* is the concentration of the protein, *P*_1_ is set to 1 and *P*_2_ is K_d_. For EMSA with RNA competition, protein was incubated with cold RNA competitors prior to the adding of RNA probes.

### m^6^A-seq

m^6^A immunoprecipitation and library construction procedure were modified from published procedure.^3^ In brief, fragmented and ethanol precipitated mRNA (6 μg) from 4-week-old mouse brain was incubated with 12 μg of anti-m^6^A polyclonal antibody (Synaptic Systems, 202003) in IPP buffer (150 mM NaCl, 0.1% NP-40, and 10 mM Tris-HCl [pH 7.4]) for 2 h at 4 °C. The mixture was then immunoprecipitated by incubation with 80 μl protein A beads (Sigma, P9424) at 4 °C for an additional 2 h. After being washed three times, bound RNA was eluted from the beads with 0.5 mg/ml N6-methyladenosine (BERRY & ASSOCIATES, PR3732) in IPP buffer and then extracted by Trizol. The remaining RNA was re-suspended in H_2_O and used for library generation with mRNA sequencing kit (Illumina).

### m^6^A-qRT-PCR

Purified mRNAs were prepared as described above and broken down into ~ 300 nt fragments by RNA Fragmentation Reagents (Ambion, AM8740) for 30 s at 94 °C. Immunoprecipitation was performed using anti-m^6^A antibody (Synaptic Systems, 202003) as described above. The enrichment of m^6^A was measured with quantitative Reverse Transcription Polymerase Chain Reaction (qRT-PCR). Primers for m^6^A-qRT-PCR (*Olig2*-peak) are listed in Supplementary information, Table S10.

### RIP-seq of Prrc2a in brain tissue

The procedure was adapted from the previous report.^16^ Three 4-week-old C57BL/6 J male mice brains were dissected and placed in 10 ml lysis buffer (150 mM NaCl, 10 mM HEPES pH 7.6, 2 mM EDTA, 0.5% NP-40, 0.5 mM DTT, 1:100 protease inhibitors cocktail, 400 U/ml RNase inhibitor). Then tissues were cut into small pieces by scissors and homogenized by homogenizer (Precellys Evolution, Bertin Technologies, Paris, France), and then the lysate was incubated on ice for 30 min. Lysates were centrifuged at 13,000× rpm for 60 min at 4 °C. Protein concentration was determined using Coomassie Plus^TM^ Protein Assay Reagent (Thermo Scientific) and diluted to final concentration at 4.0 mg/ml. 250 μl tissue lysate was saved as input by mixing with 1 ml TRIZOL and 15 ml lysate was subjected to preclear with 12 μg normal goat IgG and 150 μl protein G magnetic beads at 4 °C for 2 h. Following removal of the beads, lysates were incubated with 250 μl of protein G magnetic beads conjugated 20 μg anti-Prrc2a antibody (Sc-78859, Santa Cruz) overnight at 4 °C with rotation. The beads were collected and washed eight times with 1 ml ice-cold NT2 buffer (200 mM NaCl, 50 mM HEPES pH 7.6, 2 mM EDTA, 0.05% NP-40, 0.5 mM DTT, 400Uml^-1^ RNase inhibitor). Then the beads were resuspended in 500 μl 1×Micrococcal nuclease buffer with 2Uml^-1^ micrococcal nuclease and the mixture was incubated for 10 min at 37 °C with rotation. Collected beads were washed twice with 1×PNK + EGTA buffer (50 mM Tris-HCl pH 7.5, 20 mM EGTA pH 8.0, 0.5% NP-40) and then washed twice with NT2 buffer and once with 1×PK buffer (100 mM Tris-HCl pH 7.5, 50 mM NaCl, 10 mM EDTA, 0.2% SDS). After that, the beads were incubated with 4 mg/ml proteinase K in 200 μl 1×PK buffer for 40 min at 50 °C. RNAs recovered from supernatant by phenol-chloroform method were further subjected to rRNA removal. The purified RNAs were used to generate the library using TruSeq stranded mRNA sample preparation kit (Illumina).

### rRNA removal

The procedure was adapted from the previous report.^58^ 1 μg of fragmented RNA was dissolved in 4.5 μl hybridization buffer (200 mM NaCl, 100 mM Tris-HCl, pH 7.4). 0.5 μl of rRNA pooled oligonucleotides (1 μg) were add to dissolved RNA and the mixture was incubated in hybridization buffer at 95 °C for 2 min and then the temperature was slowly reduced (-0.5 °C/5 s) to 37 °C. 1 μl RNase H, 1 μl 10 × buffer and 3 μl NF-H_2_O was added to the mixture, which was incubated at 37 °C for 60 min and then placed on ice. We then added 1 µl DNase I and 1.2 µl 10 × DNase I buffer to the mixture and incubated the samples at 37 °C for 45 mins. We added 2.2 × RNA Clean SPRI beads to the RNase H & DNase I–treated RNA samples and mixed them by pipetting up and down 15 times. Samples were incubated on ice for 15 min or at room temperature for 5 min. The tubes were placed on an appropriate magnetic rack to separate beads from the supernatant. Beads were washed twice with 200 µl of freshly prepared 80% ethanol while in the magnetic rack. RNAs were eluted from the beads with 30 µl nuclease-free water and used for subsequent library construction.

### Sequencing data analysis

General pre-processing of reads: The m^6^A-seq, the Prrc2a RIP-seq and the RNA-seq were performed using Illumine HiSeq 3000 with paired end read length of 101–150 bp. Adaptor sequences were trimmed off for all raw reads using the Cutadapt software (version 1.10).^59^ Reads that were less than 35 nt in length or contained an ambiguous nucleotide were discarded by Trimmomatic (version 0.32).^60^ The remaining reads were aligned to the mouse genome (version mm10) using TopHat (version 2.1.0).^61^ Only uniquely-mapped reads with mapping quality score ≥ 20 were kept for subsequent analysis for each sample.

For m^6^A-seq, the m^6^A peaks in two biological replicate samples of m^6^A-immunoprecipitation were identified by MACS2 peak-calling software (version 2.1.1)^62^ with the corresponding input sample serving as control. MACS2 was run with default options except for–no model;–keep up all to turn off fragment size estimation and to keep all uniquely-mapping reads, respectively. A stringent cutoff threshold for false discovery rate (FDR) < 0.05 was used to obtain high-confidence peaks. The overlapped m^6^A peaks between two replicates were used for subsequent bioinformatic analyses.

For Prrc2a RIP-seq, the target binding regions of Prrc2a were identified using MACS2 software (version 2.1.1)^62^ with default options except for–no model. A stringent cutoff threshold for false discovery rate (FDR) < 0.05 was used to obtain high-confidence binding regions of Prrc2a.

For RNA-seq, the number of reads mapped to each Ensembl gene (release 68) was counted using the HTSeq python package (version 0.6.0),^63^ with the ‘union’ overlap resolution mode and -stranded = no. The expressions of transcripts were quantified as reads per kilobase of exon model per million mapped reads (RPKM).

### Motif identification within m^6^A and Prrc2a peaks

The motifs enriched in m^6^A peaks and Prrc2a were analyzed by HOMER (v4.7).^64^ Motif length was restricted to 5–8 nucleotide. All peaks mapped to mRNAs were used as the target sequences and background sequences were constructed by randomly shuffling peaks upon total mRNAs on genome using BEDTools’ shuffleBed (version 2.16.2).^65^

### Analysis of differentially expressed gene

Differentially-expressed genes between control and Prrc2a knockout were determined using the R-package DEGseq^66^ with the method MARS (MA-plot-based method with random sampling model), fold change cutoff = 1.2, FDR cutoff = 0.001.

### Gene ontology analysis

Gene Ontology (GO) analysis of genes with Prrc2a binding or with differential expression was performed using DAVID (http://david.abcc. ncifcrf.gov/).^67^ GO terms with *p* < 0.05 were determined to be statistically significant. GSEA was performed using GSEA software with 1000 gene set permutations.

### Statistical analysis

Statistical analyses were performed via one-way/two-way analysis of variance (ANOVA) followed by Bonferroni test or via a two-tailed unpaired Student’s *t-test*. The data are presented as the means ± SEM. *P < 0.05, ** *P* < 0.01, and *** *P* < 0.001 denote the significance thresholds.

### Data availability

The MASS data has been deposited in peptideatlas (http://www.peptideatlas.org) under access number PASS01065. The RIP-seq, RNA-seq, and m^6^A-seq data have been deposited in the Gene Expression Omnibus (GEO) database under accession numbers GSE100490, GSE100491, and GSE100492, respectively, and also deposited in GSA database (http://gsa.big.ac.cn/) under accession number PRJCA000480. All other raw data generated or analyzed during this study are included in this published paper (and its Supplementary Information Files).

## Electronic supplementary material

Supplementary information, Table S1-S10

Supplementary information, Figure S1

Supplementary information, Figure S2

Supplementary information, Figure S3

Supplementary information, Figure S4

Supplementary information, Figure S5

Supplementary information, Figure S6

Supplementary information, Figure S7

Supplementary information, Figure S8

Supplementary information, Figure S9

Supplementary information, Figure S10
