# Supplementary material for: A novel m6A reader Prrc2a controls oligodendroglial specification and myelination
Source: Cell Res. 2018 Dec 4;29(1):23–41. doi: 10.1038/s41422-018-0113-8 (PMC6318280; doi:10.1038/s41422-018-0113-8)
Supplement: Supplementary file 2 — Supplementary information, Figure S1 [file 41422_2018_113_MOESM2_ESM.pdf]

Figure S1

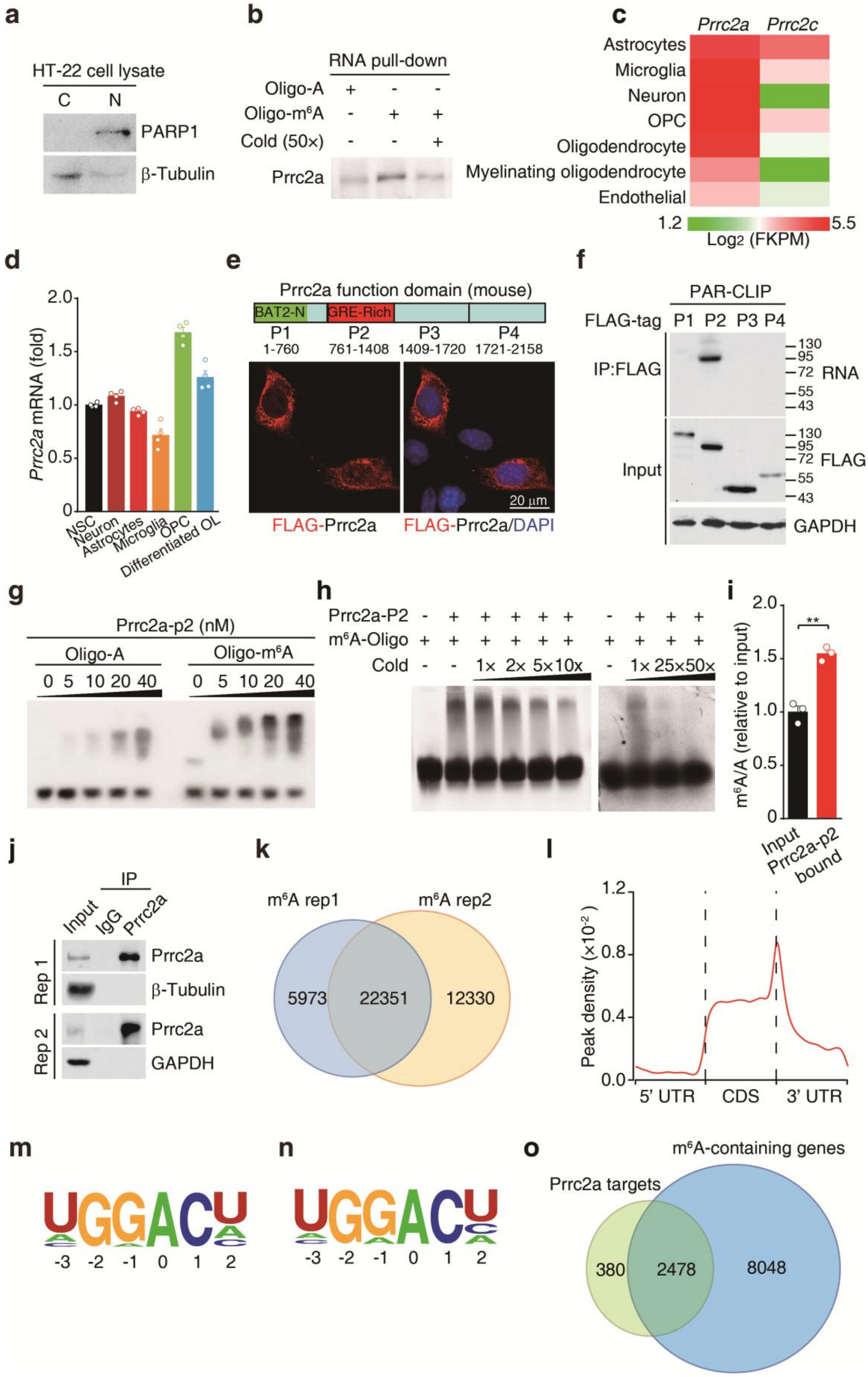

a
HT-22 cell lysate
C
N
PARP1
β-Tubulin
b
RNA pull-down
Oligo-A
Oligo-m6A
Cold (50×)
Prrc2a
c
Prrc2a
Prrc2c
Astrocytes
Microglia
Neuron
OPC
Oligodendrocyte
Myelinating oligodendrocyte
Endothelial
1.2
5.5
Log2 (FKPM)
d
Prrc2a mRNA (fold)
NSC
Neuron
Astrocytes
Microglia
OPC
Differentiated OL
e
Prrc2a function domain (mouse)
BAT2-N
GRE-Rich
P1 1-760
P2 761-1408
P3 1409-1720
P4 1721-2158
20 μm
FLAG-Prrc2a
FLAG-Prrc2a/DAPI
f
PAR-CLIP
FLAG-tag P1 P2 P3 P4
IP:FLAG
RNA
Input
FLAG
GAPDH
g
Prrc2a-p2 (nM)
Oligo-A
Oligo-m6A
0 5 10 20 40
h
Prrc2a-P2
m6A-Oligo
Cold
1× 2× 5× 10×
1× 25× 50×
i
m6A/A (relative to input)
**
Input
Prrc2a-p2 bound
j
IP
Input
IgG
Prrc2a
Rep 1
Rep 2
Prrc2a
β-Tubulin
GAPDH
k
m6A rep1
m6A rep2
5973
22351
12330
l
Peak density (×10-2)
5' UTR
CDS
3' UTR
m
UGGACU
-3 -2 -1 0 1 2
n
UGGACU
-3 -2 -1 0 1 2
o
m6A-containing genes
Prrc2a targets
380
2478
8048

1

Supplementary Figure 1, related to Figure 1. Prrc2a is an m^6A reader.

(**a**) The nuclear and cytoplasmic fractions of HT-22 cells were isolated and analyzed by western blotting using PARP1 and β-Tubulin as nuclear and cytoplasmic markers, respectively. The cytoplasmic fractions were used to identify the m^6A binding proteins (refer to Fig.1a).

(**b**) Western blotting showing Prrc2a pulled down with an m^6A-containing RNA probe, a 50 folded (50×) cold m^6A competitor were used.

(**c**) The expression of Prrc2a and Prrc2c in different types of neural cell from Brain-seq Database (http://web.stanford.edu/group/barres_lab/brain_rnaseq.html).

(**d**) RT-qPCR analyzed Prrc2a expression in different types of cultured neural cell.

(**e**) Upper panel shows schematic drawing of Prrc2a protein with predicted domains and deletion mutants and bottom panel shows immunofluorescence staining of FLAG-tagged full length Prrc2a location (red color).

(**f**) Mapping the RNA binding domains of Prrc2a. PAR-CLIP assay of RNA pulled down by Flag-tagged p1, p2, p3 and p4. RNA labeled with biotin at 3′ end was visualized by the chemiluminescent nucleic acid detection module.

(**g**) Gel-shift assay measuring the dissociation constant (K_d, nM) of His_6-Prrc2a-p2-FLAG protein with methylated and unmethylated RNA probes. 300 nmol RNA probe was labelled with biotin and the protein concentration ranged from 0 nM to 40 nM. The dissociation constant K_d(Oligo-A)=22.8 ±2.3 and K_d(Oligo-m^6A)=6.1 ±1.2.

(**h**) Labeled RNA probe and Prrc2a-p2 protein were subjected to gel-shift assay, 1 to 50 fold cold m^6A were used as a competitor.

(**i**) LC-MS/MS showing m^6A enrichment in His_6-Prrc2a-p2-FLAG bound mRNA portion (two-tailed unpaired student's *t*-test, $**P<0.01$, n=3, technical replicates).

(**j**) Western blotting detected Prrc2a immunoprecipitation efficiency of Prrc2a RIP assay in whole brain tissue at P28.

(**k**) Overlap of two biological replicates for the m^6A-seq data. Numbers are total m^6A peaks identified in each sample from whole brain at P28.

(**l**) Distribution of overlapped m^6A peaks between two biological replicates across the length of mRNA. Each region of 5' UTR, CDS, and 3' UTR were binned into 100 segments, and the percentage of m^6A-containing Prrc2a peaks that fall within each bin were determined.

(**m**) Enriched motif identified by HOMER with overlapped m^6A peaks between two biological replicates (p = 1e-200).

(**n**) Binding motif identified by HOMER with Prrc2a peaks overlapped with m^6A peaks (p = 1e-51).

(**o**) Overlap of Prrc2a binding targets and m^6A-containing genes.
