# Supplementary material for: A novel m6A reader Prrc2a controls oligodendroglial specification and myelination
Source: Cell Res. 2018 Dec 4;29(1):23–41. doi: 10.1038/s41422-018-0113-8 (PMC6318280; doi:10.1038/s41422-018-0113-8)
Supplement: Supplementary file 3 — Supplementary information, Figure S2 [file 41422_2018_113_MOESM3_ESM.pdf]

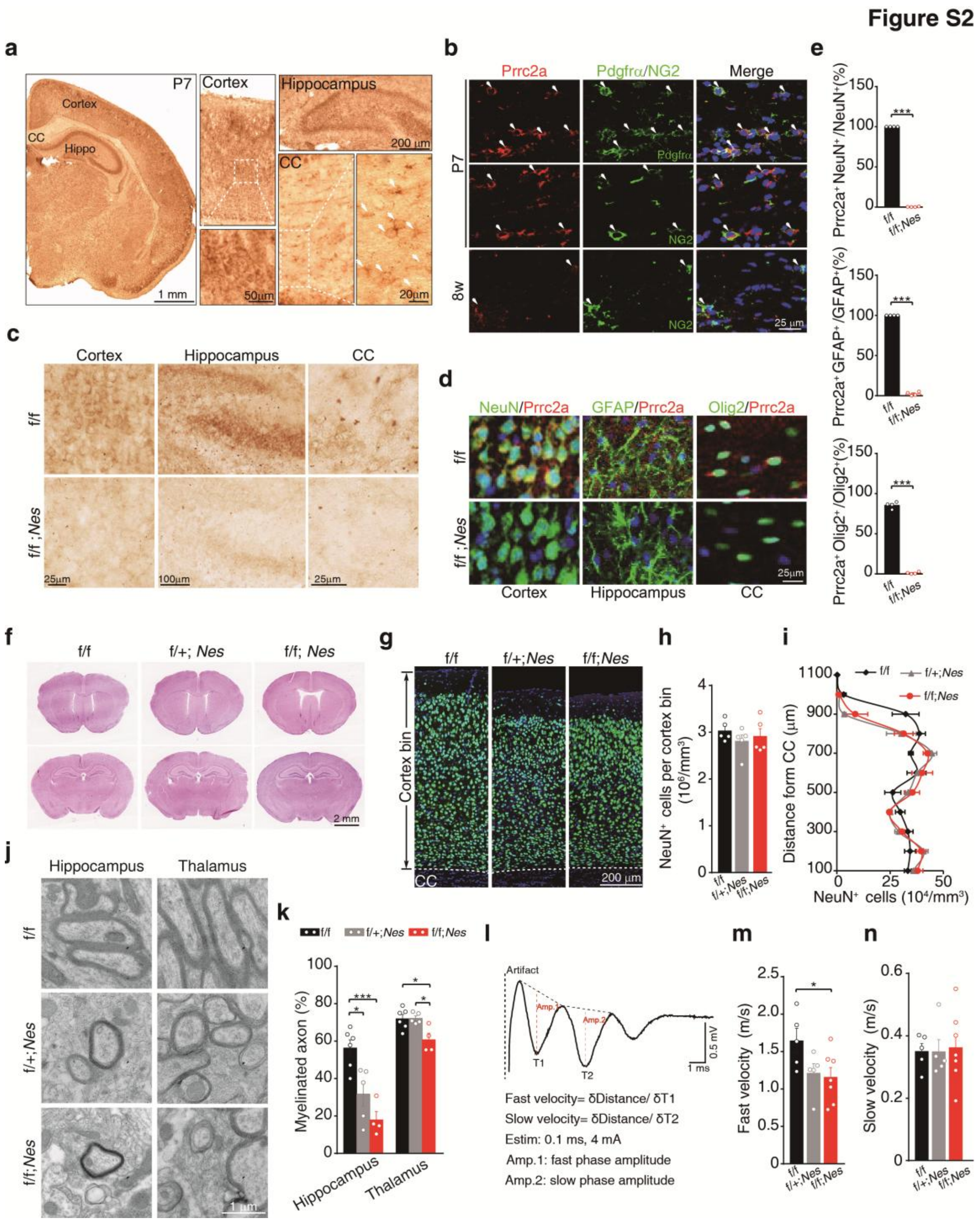

Supplementary Figure 2, related to Figure 2. Prrc2a deficiency led to hypomyelination.

(**a**) Immunohistochemical distribution of Prrc2a in a coronal section of P7 male C57BL/6J mouse brain. Cortex, cerebral cortex; Hippo, hippocampus; CC, corpus callosum.

(**b**) Confocal microscopy demonstrates that endogenous Prrc2a expressed in Pdgfrα or NG2 positive cells in corpus callosum from P7 or 8-week old male C57BL/6J mouse brain.

(**c**) Immunohistochemical staining of Prrc2a in a coronal section of brain from wild-type and *Prrc2a*$^{f/f}$*;Nestin-cre* mice at P7.

(**d**) Prrc2a co-immunostaining with NeuN, GFAP or Olig2 in the brain sections from wild-type and *Prrc2a*$^{f/f}$*;Nestin-cre* mice at P7.

(**e**) Quantification on the percentage of Prrc2a expression in NeuN, GFAP or Olig2 positive cells from wild-type and *Prrc2a*$^{f/f}$*;Nestin-cre* mice at P7 (two-tailed unpaired student's *t*-test, ***P<0.001, n=4 each group).

(**f**) Haematoxylin and eosin staining of indicated genotype brain sections at P28.

(**g**) NeuN staining of the indicated genotype mice at P56.

(**h**) Quantification of NeuN$^+$ cells in cortex bin (n = 5 each group).

(**i**) Quantification of the distance from NeuN$^+$ to the corpus callosum surface. Values for the number of NeuN$^+$ in each 100 μm bin represent mean ± SEM (n = 5 each group).

(**j**) Representative TEM of the myelin fibers in the hippocampus and thalamus showed reduced myelination in the indicated genotype mice at 4 weeks old.

(**k**) The percentage of myelinated axons in the hippocampus and thalamus from 4-week-old mice with indicated genotypes (one-way ANOVA followed Tukey test, *P<0.05, ***P<0.01, f/f, n=6; f/+; *Nes*, n=5; f/f; *Nes*, n=4).

(**l**) Typical corpus callosum compound action potentials (CAPs) evoked at 4 mA from wild-type mice at 8 weeks old. Fast velocity=δDistance/δT1; Slow velocity=δDistance/δT2; Amp.1 is the fast phase amplitude and Amp.2 is the slow phase amplitude.

(**m**) Fast velocity: callosal conduction velocity of the myelinated axons (one-way ANOVA followed Tukey test, *P<0.05, f/f n=5, f/+;*Nes* n=5, f/f;*Nes* n=7).

(**n**) Slow velocity: callosal conduction velocity of the unmyelinated axons (f/f n=5, f/+;*Nes* n=5, f/f;*Nes* n=7).
