# Supplementary material for: A novel m6A reader Prrc2a controls oligodendroglial specification and myelination
Source: Cell Res. 2018 Dec 4;29(1):23–41. doi: 10.1038/s41422-018-0113-8 (PMC6318280; doi:10.1038/s41422-018-0113-8)
Supplement: Supplementary file 4 — Supplementary information, Figure S3 [file 41422_2018_113_MOESM4_ESM.pdf]

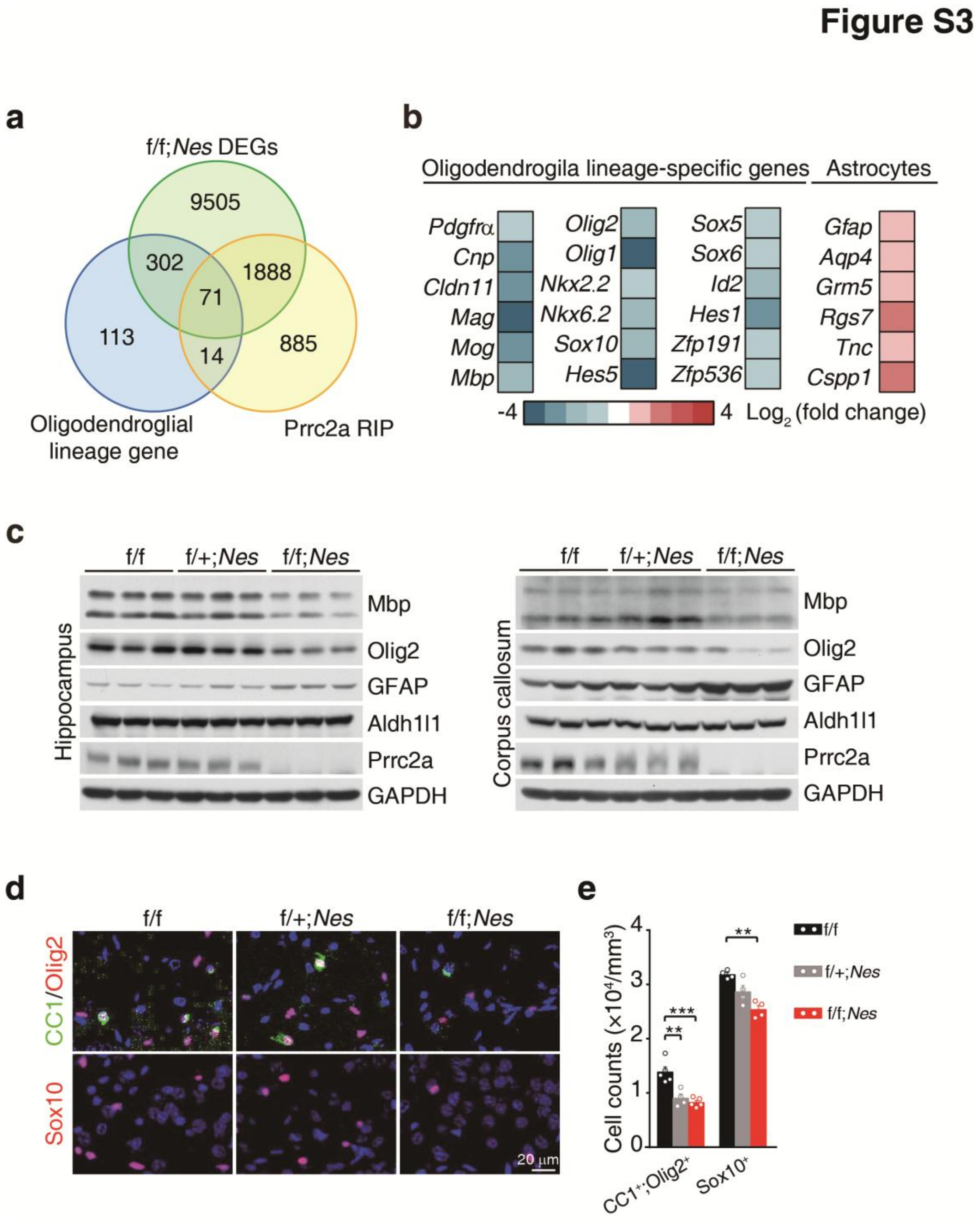

Supplementary Figure 3, related to Figure 4. Prrc2a deficiency reduces oligodendroglia developmental stage-specific marker genes.

(**a**) Overlapped oligodendroglial lineage related genes and Prrc2a targets with DEGs in *Prrc2a*$^{f/f}$; *Nestin*$^{cre+/-}$ versus control samples (P=2.81$\times10^{-14}$, hypergeometric test, see also Supplementary Table 2-4)

(**b**) Heat map depicting the representative downregulated genes related to oligodendrocytes and part of upregulated genes related to astrocytes.

(**c**) Western blot analysis of protein expression using the indicated antibody in isolated hippocampus and corpus callosum from control and $Prrc2a^{f/f};Nestin^{cre+/-}$ mice at P28.

(**d**) Immunostaining of CC1/Olig2 or Sox10 in hippocampus from mice with indicated genotypes at 4 weeks old.

(**e**) The quantification of $CC1^{+}Olig2^{+}$ or $Sox10^{+}$ cells (one-way ANOVA followed Tukey test, $^{**}P<0.01$, $^{***}P<0.001$, $CC1^{+}$ $Olig2^{+}$ cells: f/f, n=5, f/+; *Nes* n=4, f/f; *Nes* n=5, $Sox10^{+}$ cells: n=4 per group).
