# Supplementary material for: A novel m6A reader Prrc2a controls oligodendroglial specification and myelination
Source: Cell Res. 2018 Dec 4;29(1):23–41. doi: 10.1038/s41422-018-0113-8 (PMC6318280; doi:10.1038/s41422-018-0113-8)
Supplement: Supplementary file 5 — Supplementary information, Figure S4 [file 41422_2018_113_MOESM5_ESM.pdf]

Figure S4

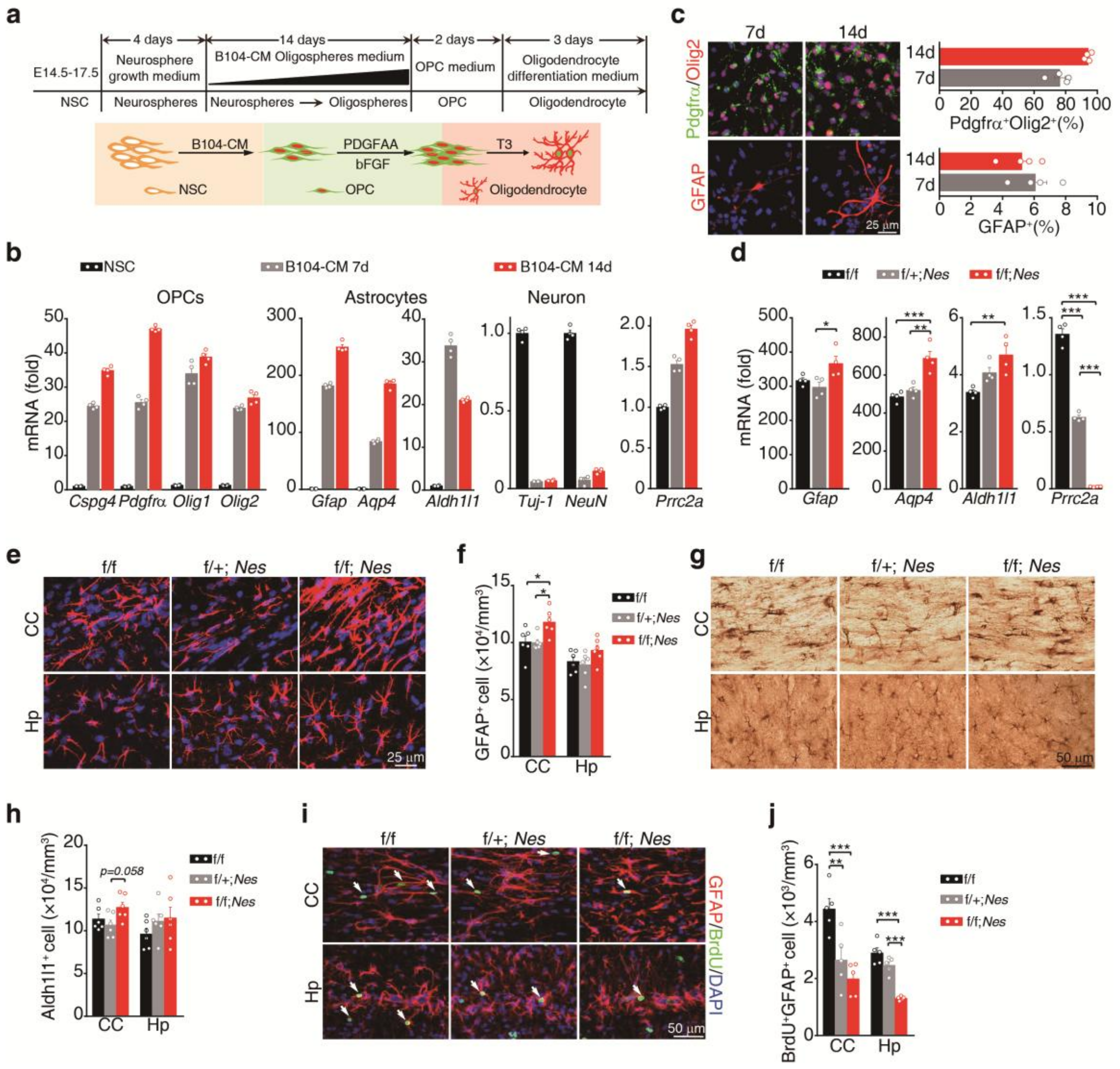

Supplementary Figure 4, related to Figure 5. Prrc2a modulates astrocytes fate determination and proliferation.

(**a**) Temporal diagram of OPC generation, proliferation and differentiation *in vitro*.

(**b**) Gene expressions during neurosphere to oligosphere transformation at 7 and 14 day after Bl04-CM treatment, respectively.

(**c**) Immunofluorescence labeling of Pdgfrα/Olig2 or GFAP in oligosphere-derived cells at 7 and 14 days after Bl04-CM treatment, respectively. The right bar graph depicts the quantification of the percentage of Pdgfrα$^+$ Olig2$^+$ and GFAP$^+$ cells.

(**d**) Gene expressions during neurosphere to oligosphere transformation in cells 14 days post B104-CM treatment. The gene expressions were normalized to those of wild-type neural stem cell (one-way ANOVA followed Tukey test, **P*<0.05, ***P*<0.01, ****P*<0.001, n=4 per group).

(**e**) Immunostaining of GFAP in corpus callosum (CC) and hippocampus (Hp) sections from indicated genotype mice at P28.

(**f**)The quantification of GFAP positive cells (one-way ANOVA followed Tukey test, **P*<0.05, n = 6 per group).

(**g**) Immunohistochemically staining of Aldh1l1 corpus callosum (CC) and hippocampus (Hp) sections from indicated genotype mice at P28.

(**h**)The quantification of Aldh1l1 positive cells (one-way ANOVA followed Tukey test, n = 6 per group).

(**i**) BrdU (50mg/kg) was intraperitoneally injected into P6 mice. 2h later, the mice were sacrificed and the brain sections of CC and Hp were immunostained with anti-GFAP and anti-BrdU antibodies. Arrowheads indicate the proliferating astrocytes (BrdU and GFAP double-positive cells).

(**j**)The quantification of the proliferating astrocytes from the indicated genotype mice (one-way ANOVA followed Tukey test, ***P*<0.01, ****P*<0.001, n=5 each group).
