# Supplementary material for: A novel m6A reader Prrc2a controls oligodendroglial specification and myelination
Source: Cell Res. 2018 Dec 4;29(1):23–41. doi: 10.1038/s41422-018-0113-8 (PMC6318280; doi:10.1038/s41422-018-0113-8)
Supplement: Supplementary file 6 — Supplementary information, Figure S5 [file 41422_2018_113_MOESM6_ESM.pdf]

Figure S5

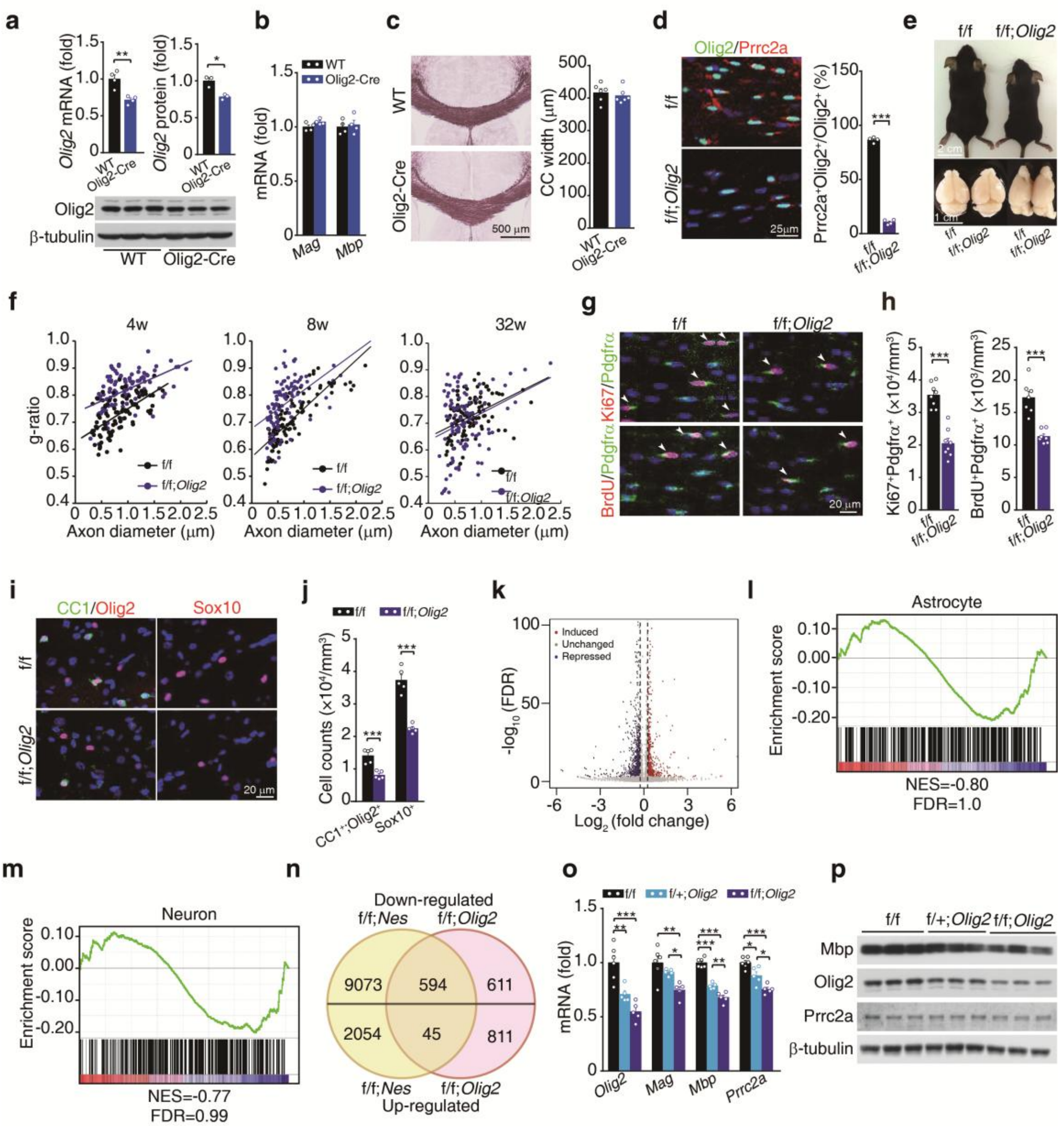

Supplementary Figure 5, related to Figure 6. Prrc2a deletion in the oligodendroglial lineage leads to hypomyelination.

(**a**) The gene (left upper panel) and protein (right upper panel and bottom panel) expression of Olig2 in brain tissues from WT and Olig2-cre mice at 8 weeks old (two-tailed unpaired Student's *t-test*, **P*<0.05, ***P*<0.01; RNA level, n=4 per group; protein level, n=3 per group).

(**b**) The *Mag* and *Mbp* expressions of brain tissues from wild-type and Olig2-cre mice at 8 weeks old (n=4 per group).

(**c**) Black gold staining of corpus callosum in brain slices from WT and Olig2-Cre mice at 4 weeks old. The quantification of corpus callosum width at the midline was shown in right bar (n=6 per group).

(**d**) Prrc2a co-immunostained with Olig2 in corpus callosum from control and *Prrc2a*$^{f/f}$*;Olig2-cre* mice at P9. The quantification of the percentage of Prrc2a expression in Olig2 positive cells (two-tailed unpaired Student's *t-test*, ****P*<0.001, n = 4 each group) was shown in the right panel.

(**e**) Representative picture of *Prrc2a*$^{f/f}$*;Olig2*$^{cre+/-}$ and control mice at P28.

(**f**) Scatterplots of the myelin g ratios of the corpus callosum at 4, 8 and 32 week-old *Prrc2a*$^{f/f}$*; Olig2*$^{cre+/-}$ and control mice (general linear model and ANCOVA analysis, 4w: *P*<0.001; 8w: *P*<0.001, 32w: *P*=0.124; More than 100 axons from each genotype and time points were analyzed).

(**g**) *Prrc2a f/f; Olig2-cre* and control mice at P9 were sacrificed and the brain sections of corpus callosum were immunostained with Pdgfrα and Ki67 (upper panel) or mice at P6 were intraperitoneally injected with 50mg/kg BrdU, and 2 hours later, the mice were sacrificed and the brain sections of corpus callosum were immunostained for Pdgfrα and BrdU (bottom panel). Arrowheads indicate the proliferating OPCs (Pdgfrα and Ki67/BrdU double-positive cells).

(**h**) The quantification of Pdgfrα$^+$Ki67$^+$ or Pdgfrα$^+$BrdU$^+$ in corpus callosum from the indicated genotype mice (two-tailed unpaired Student's *t-test*, ****P*<0.001, Pdgfrα$^+$Ki67$^+$: n=8 each group; Pdgfrα$^+$BrdU$^+$: n=7 each group).

(**i**) Immunostaining of CC1/Olig2 or Sox10 in hippocampus from 4-week-old mice with

indicated genotypes.

(**j**)The quantification of $CC1^+$ $Olig2^+$ or $Sox10^+$ cells (two-tailed unpaired Student's *t-test*, ****P*<0.001, n=5 per group).

(**k**) Volcano plot of RNA-seq data showing Prrc2a-regulated genes from brain tissue samples of 4-week-old $Prrc2a^{f/f}$*; *$Olig2^{Cre+/-}$ versus control mice.

(**l**) GSEA plots evaluating the changes in astrocyte related genes from brain tissue samples of $Prrc2a^{f/f}$*; *$Olig2^{Cre+/-}$ versus control mice.

(**m**) GSEA plots evaluating the changes in neuron related genes from brain tissue samples of $Prrc2a^{f/f}$*; *$Olig2^{Cre+/-}$ versus control mice.

(**n**) Overlapped DEGs from $Prrc2a^{f/f}$*; *$Nestin^{cre+/-}$ and $Prrc2af^{f}$*; *$Olig2^{cre+/-}$ versus their controls.

(**o**) RT-qPCR analysis of the myelin related gene expression in Prrc2a deficiency and control mice at 6 weeks old (one-way ANOVA followed Tukey test, **P*<0.05, ***P*<0.01, ****P*<0.001, f/f, n=6; f/+; *Olig2*, n=5; f/f; *Olig2*, n=5).

(**p**) Western blot analysis of protein expression using the indicated antibodies in isolated hippocampus from Prrc2a deficiency and control mice at 6 weeks old.
