# Supplementary material for: A novel m6A reader Prrc2a controls oligodendroglial specification and myelination
Source: Cell Res. 2018 Dec 4;29(1):23–41. doi: 10.1038/s41422-018-0113-8 (PMC6318280; doi:10.1038/s41422-018-0113-8)
Supplement: Supplementary file 7 — Supplementary information, Figure S6 [file 41422_2018_113_MOESM7_ESM.pdf]

**Figure S6**

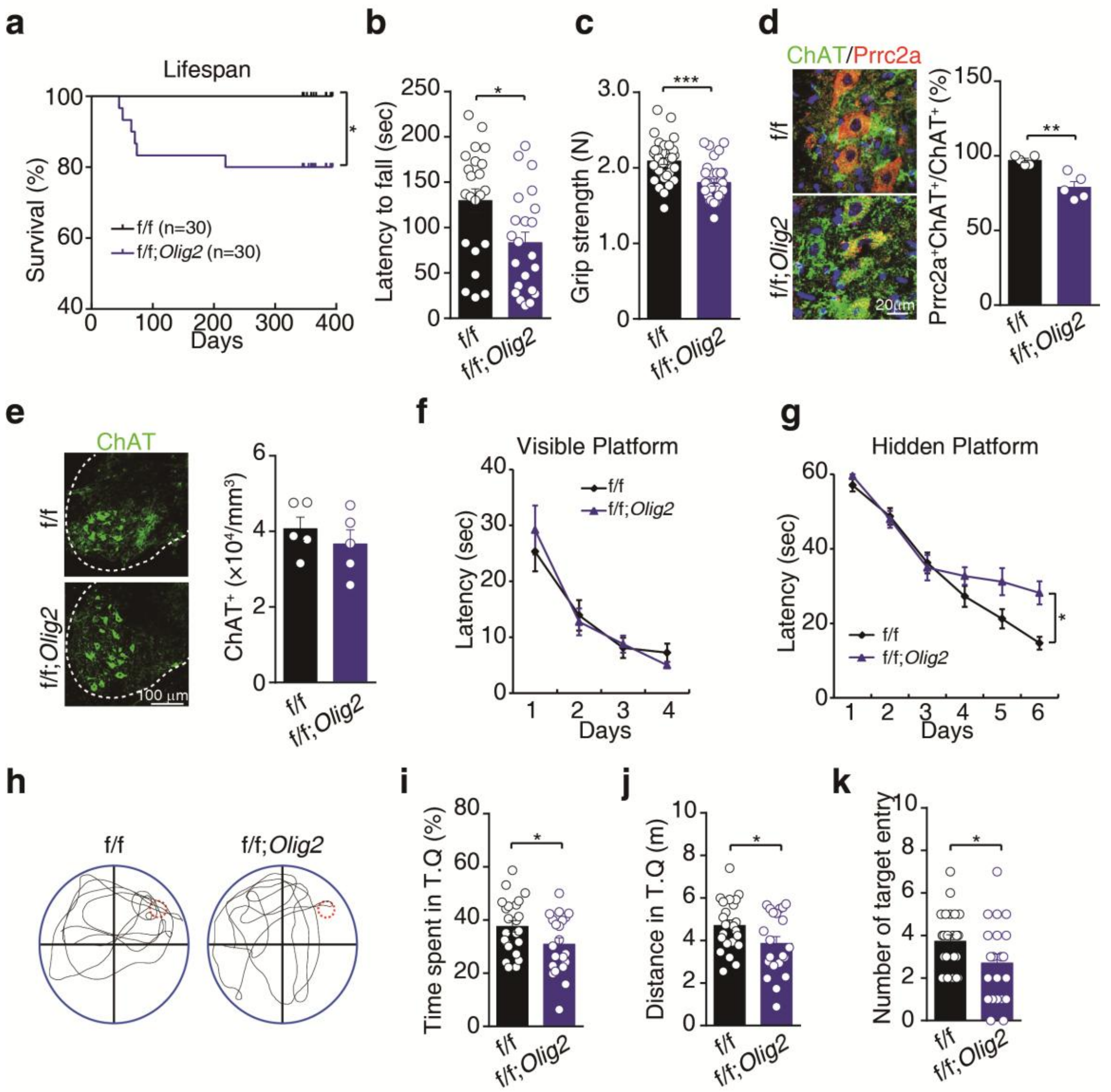


**Supplementary Figure 6, related to Figure 6. Prrc2a deletion in the oligodendroglial lineage leads to locomotive and cognitive defects.**

(**a**) Kaplan-Meier survival curves. Statistical significance was determined by the log rank test (*P*=0.0105, n=30 each group).

(**b**) The latency of 4-month-old mice with indicated genotypes on the Rota-Rod (two-tailed unpaired Student's *t-test*, **P*<0.05, n=24 per group).

(**c**) Grip strength analysis of 4-month-old mice with indicated genotypes (two-tailed unpaired Student's *t-test*, ****P*<0.001, f/f, n=36, f/f; *Olig2* n=31).

(**d**) Prrc2a co-immunostained with motor neuron marker ChAT in spinal cord from 4-week-old *Prrc2a*$^{f/f}$*; Olig2*$^{Cre+/-}$ and control mice. The percentage of Prrc2a expression in ChAT positive neuron was shown in the right bar graph (two-tailed unpaired Student's *t-test*, **$P<0.01$, n=5 per group).

(**e**) The representative ChAT immunochemistry staining pictures (left) and the quantification of motor neurons (right) from spinal cords of 4-month-old *Prrc2a*$^{f/f}$ and *Prrc2a*$^{f/f}$*; Olig2*$^{Cre+/-}$ mice (two-tailed unpaired Student's *t-test*, $P=0.4304$, n=5 per group).

(**f**) The mean escape latency (±SEM) for mice to reach the platform in the visible version of the water maze is plotted against the day of the experiment. ($P=0.9052$, $F=0.01434$; Two-way ANOVA followed by Bonferroni test. f/f n=23, f/f; *Olig2* n=21).

(**g**) The mean escape latency (±SEM) for mice to reach the platform in the hidden version of the water maze is plotted against the day of the experiment ($P=0.0137$, $F=6.643$; Two-way ANOVA followed by Bonferroni test. f/f n=23, f/f; *Olig2* n=21).

(**h**) Probe trial was performed 24 hours after the last training session by removing the platform. Probe represents vertical view of the tracks of indicated genotype mice.

(**i**) Time spent in the target quadrant during probe trial (two-tailed unpaired Student's *t-test*, *$P<0.05$, f/f n=23, f/f; *Olig2* n=21).

(**j**) Traveled distance in the target quadrant during probe trial (two-tailed unpaired Student's *t-test*, *$P<0.05$, f/f n=23, f/f; *Olig2* n=21).

(**k**) The number of platform crossing from the same group of mice tested in the probe trial (two-tailed unpaired Student's *t-test*, *$P<0.05$, f/f n=23, f/f; *Olig2* n=21).

The water maze behavior was tested in 6 month-old mice.
