# Supplementary material for: A novel m6A reader Prrc2a controls oligodendroglial specification and myelination
Source: Cell Res. 2018 Dec 4;29(1):23–41. doi: 10.1038/s41422-018-0113-8 (PMC6318280; doi:10.1038/s41422-018-0113-8)
Supplement: Supplementary file 8 — Supplementary information, Figure S7 [file 41422_2018_113_MOESM8_ESM.pdf]

Figure S7

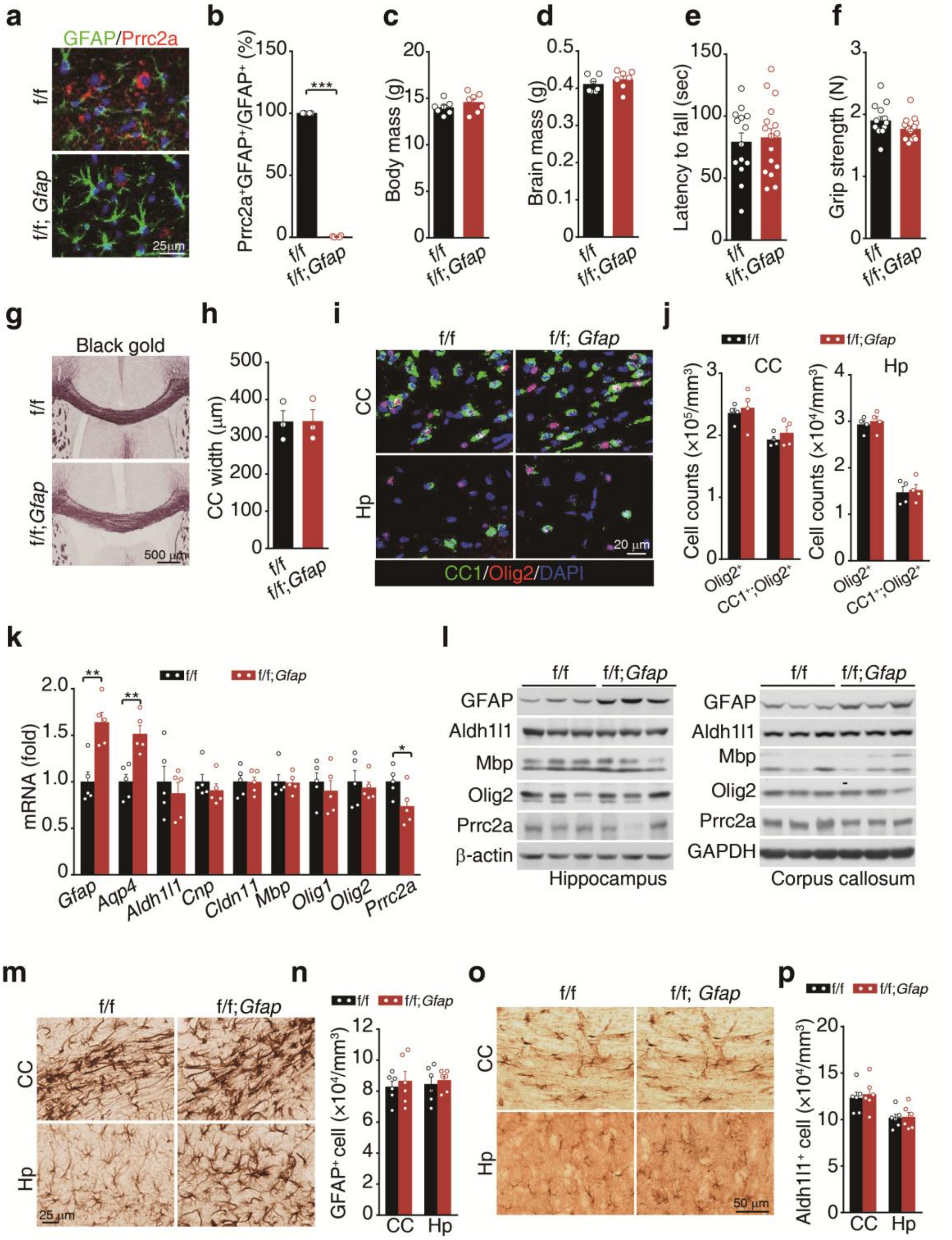

Supplementary Figure 7. Astrocytic Prrc2a deletion does not affect myelination.

(**a**) Prrc2a co-immunostained with astrocyte marker GFAP in hippocampus from 4-week-old *Prrc2a*$^{f/f}$*; Gfap*$^{Cre+/-}$ and control mice.

(**b**) The percentage of Prrc2a expression in GFAP positive cells (two-tailed unpaired Student's

t-test, ***P<0.001, n=4 per group).

(**c**, **d**) The body and whole wet brain mass of *Prrc2a*$^{f/f}$;*Gfap*$^{cre+/-}$ and control mice at P28 (two-tailed unpaired Student's *t-test*, Body mass, P=0.2760; Brain mass, P=0.3220, n=7 each group).

(**e**) The latency of 3-month-old mice on the Rota-Rod. (two-tailed unpaired Student's *t-test*, P=0.7399; f/f, n=14, f/f; *Gfap* n=16).

(**f**) Grip strength analysis of 3-month-old mice with indicated genotypes (two-tailed unpaired Student's *t-test*, P=0.0975, f/f, n=14, f/f; *Gfap* n=16).

(**g**) Black gold staining of brain slices from 4-weeks-old *Prrc2a*$^{f/f}$; *Gfap*$^{cre+/-}$ and control mice.

(**h**) The quantification of corpus callosum width at the midline (two-tailed unpaired Student's *t-test*, P=0.9836, n=3 per group).

(**i**) Immunostaining of CC1/Olig2 or Sox10 in CC and Hp from 4-week-old mice with indicated genotypes. The quantification of Olig2$^+$, CC1$^+$ Olig2$^+$ or Sox10$^+$ cells was shown in (**j**) (two-tailed unpaired Student's *t-test*, Olig2$^+$ cells in CC, P=0.6595; CC1$^+$ Olig2$^+$ cells in CC, P=0.4004; Olig2$^+$ cells in Hp, P=0.6393; CC1$^+$ Olig2$^+$ cells in Hp, P=0.7911; n=4 per group).

(**k**) Quantitative PCR analysis of the level of mRNA in isolated hippocampus of control and *Prrc2a*$^{f/f}$;*Gfap*$^{cre+/-}$ mice at 4 weeks old (two-tailed unpaired Student's *t-test*, *P<0.05, **P<0.01, n=5 per group).

(**l**) Western blotting analysis of the protein expression using the indicated antibodies in isolated hippocampus and corpus callosum from control and *Prrc2a*$^{f/f}$;*Gfap*$^{cre+/-}$ mice at P28.

(**m**) Immunochemistry staining of GFAP in CC and Hp sections from indicated genotype mice

at P28. The quantification of GFAP positive cells was shown in (**n**) (two-tailed unpaired Student's *t-test*, CC: *P*=0.6321; Hp: *P*=0.6495; n = 6 per group).

(**o**) Immunohistochemically staining of Aldh1l1 in CC and Hp sections from indicated genotype mice at P28. The quantification of Aldh1l1 positive cells was shown in (**p**) (two-tailed unpaired Student's *t-test*, CC: *P*=0.6843; Hp: *P*=0.8719; n = 6 per group).
