# Supplementary material for: A novel m6A reader Prrc2a controls oligodendroglial specification and myelination
Source: Cell Res. 2018 Dec 4;29(1):23–41. doi: 10.1038/s41422-018-0113-8 (PMC6318280; doi:10.1038/s41422-018-0113-8)
Supplement: Supplementary file 9 — Supplementary information, Figure S8 [file 41422_2018_113_MOESM9_ESM.pdf]

Figure S8

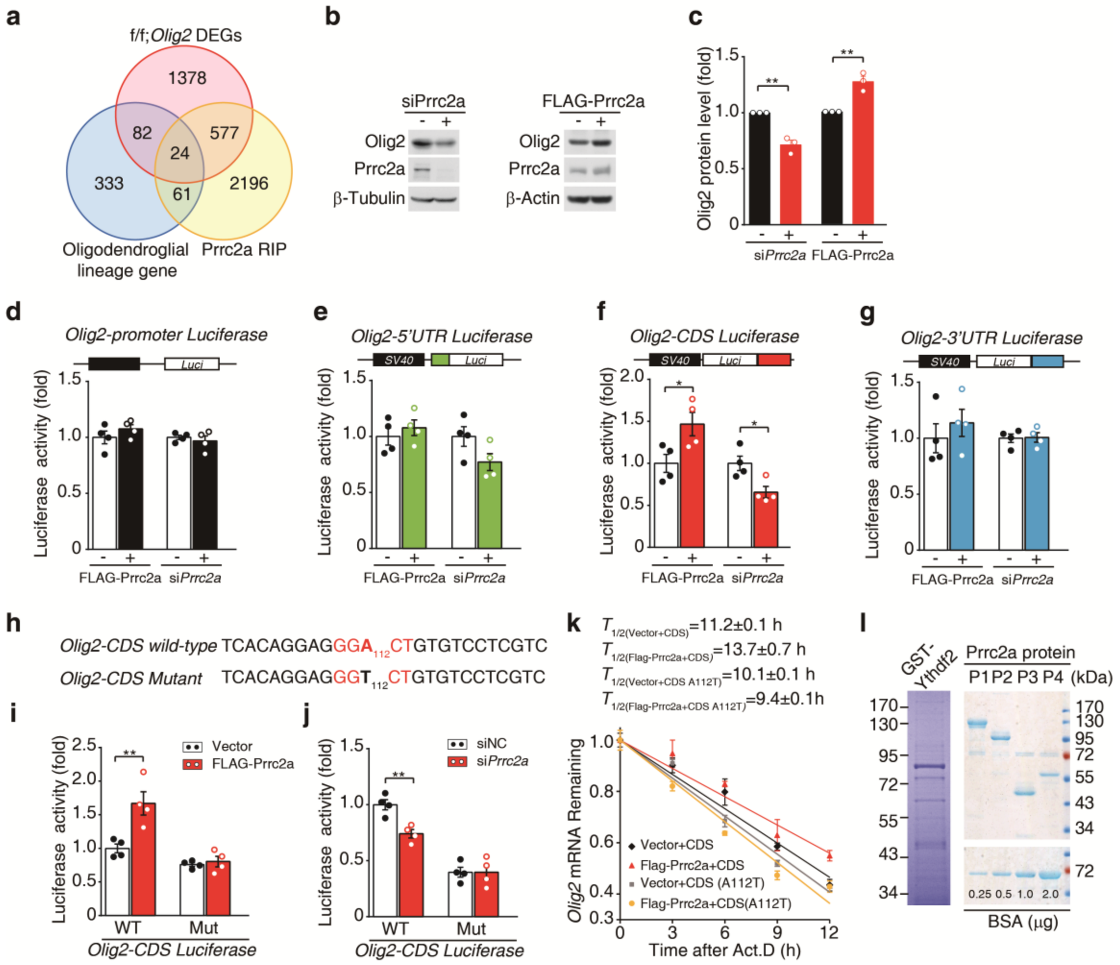


**Supplementary Figure 8, related to Figure 7. Prrc2a regulates *Olig2* mRNA in an m$^6$A-dependent manner.**

(**a**) Venn diagram of oligodendroglial lineage related genes and Prrc2a targets overlapped with DEGs from *Prrc2a*$^{f/f}$; *Olig2*$^{cre+/-}$ versus control samples (see also Supplementary Table 7).

(**b**) GL261 cells were transfected with or without Prrc2a siRNA for 72 h (left) or FLAG-tagged Prrc2a (right). Lysates of cells were immunoblotted with indicated antibodies.

(**c**) The quantitation of Olig2 protein levels in (**b**) (two-tailed unpaired student's *t*-test, **$P$<0.01; n=3 independent assay).

(**d**) Olig2 promoter luciferase activity was analyzed in GL261 cells with Prrc2a overexpression or knockdown (two-tailed unpaired student's *t*-test, vector vs. FLAG-Prrc2a,

*P*=0.3062; si*NC* vs. si*Prrc2a*, *P*=0.5409, n=4 per group).

(**e**) Olig2 5'-UTR luciferase activity was analyzed in GL261 cells with Prrc2a overexpression or knockdown (two-tailed unpaired student's *t*-test, vector vs. FLAG-Prrc2a, *P*=0.4820; si*NC* vs. si*Prrc2a*, *P*=0.0971; n=4 per group).

(**f**) Olig2 coding sequence (CDS) luciferase activity was analyzed in GL261 cells with Prrc2a overexpression or knockdown (two-tailed unpaired student's *t*-test, **P*<0.05, n=4 per group).

(**g**) Olig2 3'UTR luciferase activity was analyzed in GL261 cells with Prrc2a overexpression or knockdown (two-tailed unpaired student's *t*-test, vector vs. FLAG-Prrc2a, *P*=0.4710; si*NC* vs. si*Prrc2a*, *P*=0.9071; n=4 per group).

(**h**) Combined $m^6A$-seq data with SRAMP software analysis identified a very high confident $m^6A$ site ($GG\mathbf{A}_{112}CT$, a conserved $m^6A$ methylation motif) in *Olig2 CDS* region.

(**i**) Luciferase activities of wild-type Olig2-CDS or mutant Olig2-CDS (A112T) were analyzed in GL261 cells with Prrc2a overexpression (one-way ANOVA followed by Tukey test, ***P*<0.01, n=4 per group).

(**j**) Wild-type Olig2-CDS or mutant Olig2-CDS (A112T) luciferase activity was analyzed in GL261 cells with Prrc2a knockdown (one-way ANOVA followed by Tukey test, ***P*<0.01, n=4 per group).

(**k**) At 24 h after transfection with a vector expressing Prrc2a or the control vector together with FLAG-tagged wild-type or A112T mutant Olig2 coding sequence (Olig2-CDS), GL261 cells were exposed to actinomycin D (2 μg/ml), then RNA was isolated at indicated time points. RT-qPCR was performed to assess the half-lives of Olig2-CDS. The data were presented as means ± s.e.m. and the inserted numbers ($T_{1/2\ (vetor+CDS)}$=11.1±0.1h;

$T_{1/2(\text{FLAG-Prrc2a+CDS})}$ =13.7 ±0.7h; $T_{1/2\ (\text{vetor+CDS A112T})}$=10.1 ±0.1h; $T_{1/2(\text{FLAG-Prrc2a+CDS A112T})}$ =9.4 ±0.1h) indicated the calculated half-life time from four independent experiments.

(**l**) Coomassie brilliant blue staining of the purified GST-Ythdf2 and FLAG tagged Prrc2a P1-P4 protein.
