# Supplementary material for: A novel m6A reader Prrc2a controls oligodendroglial specification and myelination
Source: Cell Res. 2018 Dec 4;29(1):23–41. doi: 10.1038/s41422-018-0113-8 (PMC6318280; doi:10.1038/s41422-018-0113-8)
Supplement: Supplementary file 10 — Supplementary information, Figure S9 [file 41422_2018_113_MOESM10_ESM.pdf]

Figure S9

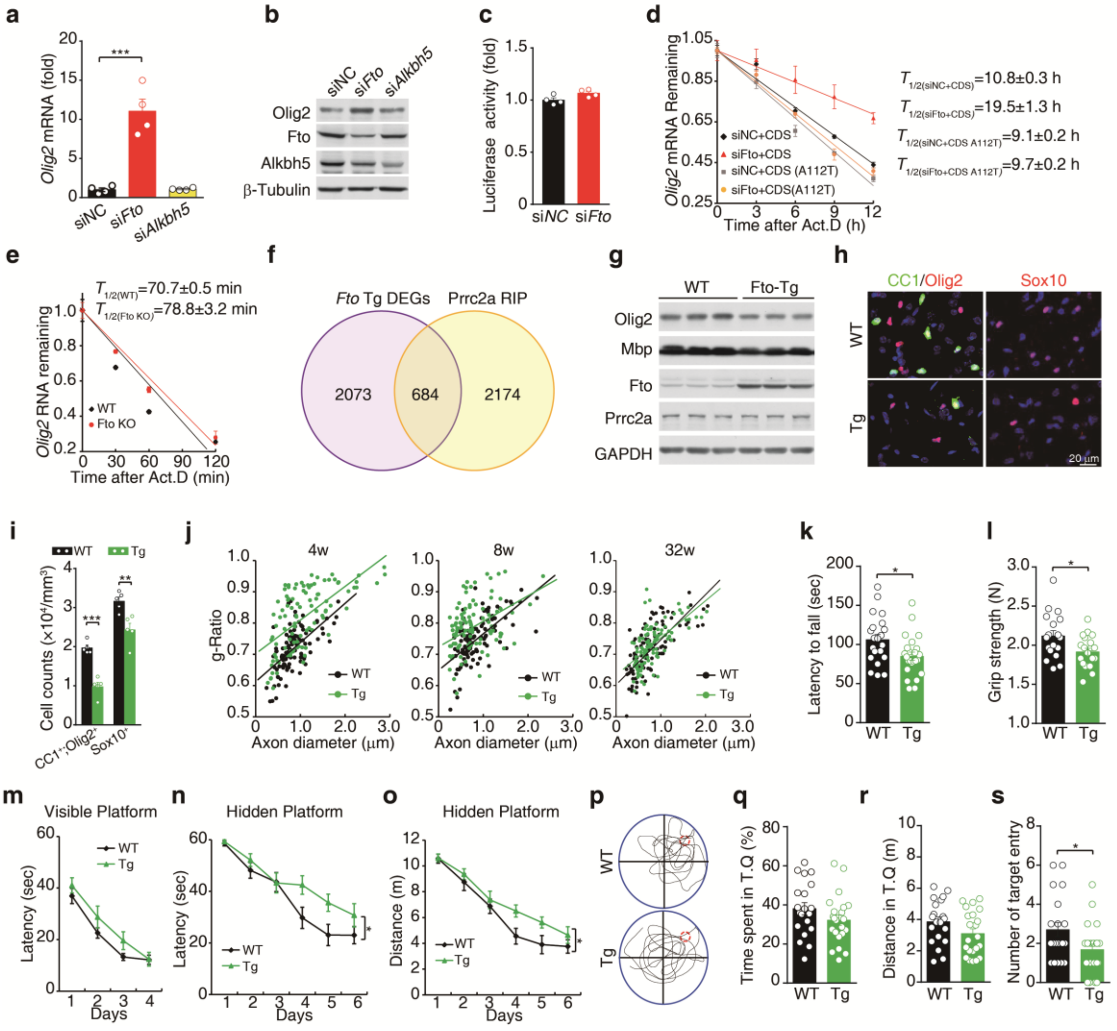

Supplementary Figure 9, related to Figure 8. Fto transgene reduces oligodendroglia population and induces behavior defects.

(**a**) The mRNA level of *Olig2* in GL261 cell treated with control, *Fto* or *Alkbh5* siRNA (one-way ANOVA followed by Tukey test, ***P<0.001, n=4 per group).

(**b**) GL261 cells were treated with control, Fto or Alkbh5 siRNA for 72h and the cell lysates were immunoblotted with indicated antibodies.

(**c**) After GL261 cells were transfected with control siRNA or Fto siRNA. 48 hours later, the cells were then transfected with Olig2 promoter luciferase plasmid. Olig2 promoter luciferase activities were analyzed 24 hours later (two-tailed unpaired student's t-test, P=0.0560, n=4

per group).

(**d**) At 72 h after transfection with Fto siRNA or control together with wild-type or FLAG-tagged Olig2 A122T mutant coding sequence (Olig2-CDS), GL261cells were exposed to actinomycin D (2 μg/ml), then RNA was isolated at indicated time points. RT-qPCR was performed to assess the half-lives of Olig2-CDS. The data were presented as means ± s.e.m. and the inserted numbers ($T_{1/2\ (siNC+CDS)}$=10.8±0.3h; $T_{1/2(siFto+CDS)}$ =19.5±1.3h; $T_{1/2\ (siNC+CDS\ A112T)}$=9.1±0.2h; $T_{1/2\ (siFto\ +CDS\ A112T)}$ =9.7±0.2h) indicated the calculated half-life time from four independent experiments.

(**e**) Cultured OPCs from wild-type or Fto knockout (KO) mice were exposed to actinomycin D (1 μg/ml), then RNA was isolated at indicated time points. RT-qPCR was performed to assess the half-life time of *Olig2* mRNA. The data were presented as means ± s.e.m. and the inserted numbers ($T_{1/2\ (WT)}$ =70.7±0.5 min; $T_{1/2(KO)}$ =78.8±3.2 min) indicated the calculated half-life time from four independent experiments.

(**f**) Overlap of Prrc2a target genes and DEGs from brain tissue samples of *Fto* transgenic versus control mice (see also Supplementary Table 9).

(**g**) Western blot analysis of protein expression using indicated antibodies in isolated hippocampus of wild-type (WT) and *Fto* transgenic mice (Tg) at P28.

(**h**) Immunostaining of CC1/Olig2 or Sox10 in hippocampus from mice with indicated genotypes at 4 weeks old. The quantification of $CC1^{+}$ $Olig2^{+}$ or $Sox10^{+}$ cells was shown in (**i**) (two-tailed unpaired student's *t*-test, $^{**}P<0.01$, $^{***}P<0.001$, n=5 per group).

(**j**) Scatterplots of the myelin g ratios of the corpus callosum from 4-, 8- and 32-week-old Fto Tg and WT mice (general linear model and ANCOVA analysis, 4w: $P<0.001$; 8w: $P<0.001$,

32w: $P=0.176$; More than 100 axons from each genotype and time points were analyzed).

(**k**) The latency of the 2-month-old mice on the Rota-Rod (two-tailed unpaired student's t-test, *$P<0.05$; WT, n=21, Tg n=24).

(**l**) Grip strength analysis of at 2-month-old mice with indicated genotypes (two-tailed unpaired student's t-test, *$P<0.05$; WT, n=18, Tg n=20).

(**m**) The mean escape latency (±SEM) for mice to reach the platform in the visible version of the water maze is plotted against the day of the experiment ($P=0.2171$, $F=1.573$; Two-way ANOVA followed by Bonferroni test, WT, n=20, Tg n=22).

(**n**) The mean escape latency (±SEM) for mice to reach the platform in the hidden version of the water maze is plotted against the day of the experiment ($P=0.0244$, $F=5.475$; Two-way ANOVA followed by Bonferroni test. WT, n=20, Tg n=22).

(**o**) The mean traveled distance (±SEM) for mice to reach the platform in the hidden version of the water maze is plotted against the day of the experiment ($P=0.0158$, $F=6.352$; Two-way ANOVA followed by Bonferroni test. WT, n=20, Tg n=22).

(**p**) Probe trial was performed 24 hours after the last training session by removing the platform. Probe represents vertical view of the tracks of indicated genotype mice.

(**q**) Time spent in the target quadrant during probe trial (two-tailed unpaired student's t-test, $P=0.1613$; WT, n=20, Tg n=22).

(**r**) Traveled distance in the target quadrant during probe trial (two-tailed unpaired student's t-test, $P=0.0874$; WT, n=20, Tg n=22).

(**s**) The number of platform crossing from the same group of mice tested in the probe trial (two-tailed unpaired student's t-test, $P=0.0311$; WT, n=20, Tg n=22).

The water maze behavior was tested in 3 month-old mice.
