# Supplementary material for: A novel m6A reader Prrc2a controls oligodendroglial specification and myelination
Source: Cell Res. 2018 Dec 4;29(1):23–41. doi: 10.1038/s41422-018-0113-8 (PMC6318280; doi:10.1038/s41422-018-0113-8)
Supplement: Supplementary file 11 — Supplementary information, Figure S10 [file 41422_2018_113_MOESM11_ESM.pdf]

Figure S10

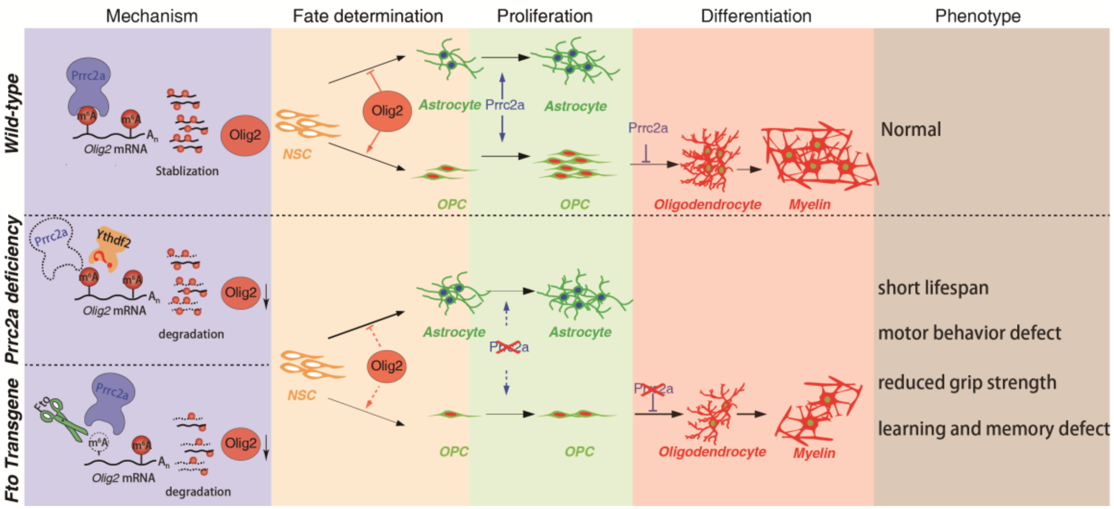

Supplementary Figure 10. Model depicting the role of Prrc2a and Fto in oligodendroglial specification through modulating m^{6}A modification of *Olig2* mRNA.
